# PBPK Modeling of CES1-Activated Prodrugs Across Different Age Groups: Simultaneous Prodrug–Metabolite Prediction for Oseltamivir, Enalapril, and Cilazapril

**DOI:** 10.3390/pharmaceutics18101211

**Published:** 2026-09-23

**Authors:** Ping Li, Yiting Yang, Ling Jiang, Jiawei Wang

**Affiliations:** 1 Department of Pharmacy, Beijing Tongren Hospital, Capital Medical University, Beijing 100730, China; lplh2023@163.com; 2 Hangzhou Institute of Medicine (HIM), Zhejiang Cancer Hospital, Chinese Academy of Sciences, Hangzhou 310022, China; yangyt3175@zjcc.org.cn; 3 Center of Drug Metabolism and Pharmacokinetics, School of Pharmacy, China Pharmaceutical University, Nanjing 210009, China; jiangling0620208@163.com

**Keywords:** physiologically based pharmacokinetic model, carboxylesterase 1, prodrug, pediatric population, geriatric population

## Abstract

**Background/Objectives**: Carboxylesterase 1 (CES1) is a key enzyme involved in the bioactivation of ester prodrugs. This study aimed to construct a mechanistic, whole-body, physiologically based pharmacokinetic (PBPK) model to simultaneously predict the pharmacokinetics of CES1-activated prodrugs and their metabolites across different age groups. **Methods**: A comprehensive whole-body prodrug–metabolite PBPK framework was constructed by integrating age-specific physiological parameters. Oseltamivir and enalapril were evaluated across adult, pediatric, and geriatric populations, whereas cilazapril validation was focused on adult and geriatric cohorts based on clinical data availability. The model was developed and validated in adults, and then extrapolated to pediatric and geriatric populations. Model performance was evaluated by comparing predicted and observed pharmacokinetic parameters, specifically the area under the curve (${AUC}_{0-t}$) and maximum plasma concentration ($C_{max}$), with an acceptance criterion of 0.5- to 2.0-fold error. **Results**: The majority of observed plasma concentrations fell within the 5th–95th percentiles of predicted profiles. Additionally, for most simulations, the ${AUC}_{0-t}$ and $C_{max}$ were within 0.5- to 2-fold of the observed values. Sensitivity analyses revealed that the exposure of their active metabolites was highly related to the plasma unbound fraction, glomerular filtration rate, and hepatic CES1-mediated bioactivation. **Conclusions**: This whole-body PBPK framework provides a mechanistic basis for predicting CES1-mediated prodrug disposition across age groups. The framework achieved clinical validation across pediatric, adult, and geriatric populations for oseltamivir and enalapril, and provided proof-of-concept support for extending the approach to cilazapril. This platform may serve as a basis for future pharmacokinetic/pharmacodynamic integration and model-informed dose optimization.

## 1. Introduction

Pediatric and geriatric populations exhibit distinct physiological characteristics that significantly influence pharmacotherapy. Age-related differences in gastrointestinal motility, organ function, and enzymatic activity lead to altered pharmacokinetic profiles. Although population pharmacokinetic models have provided dosing recommendations for various age groups using empirical scaling methodologies, these ‘top-down’ approaches often lack mechanistic insights to capture age-related physiological variability [1]. In contrast, physiologically based pharmacokinetic (PBPK) models integrate physiological and substance-specific properties, enabling a systematic and mechanistic exploration of pharmacokinetics across diverse populations. PBPK models have been successfully applied to predict pharmacokinetics for drugs metabolized by cytochrome P450 enzymes and UDP-glucuronosyltransferases [2,3,4], and they are increasingly endorsed by regulatory agencies to support dose selection in vulnerable populations [5,6].

Carboxylesterase 1 (CES1) is the predominant hepatic phase I drug-metabolizing enzyme, accounting for 80–95% of the total hydrolytic activity in the human liver [7,8]. It is crucial for the activation of ester prodrugs, such as oseltamivir and several angiotensin-converting enzyme inhibitors (ACEIs). Notably, CES1 expression and functionality are age-dependent, potentially affecting pharmacokinetics and pharmacodynamics in pediatric patients. Significantly lower hepatic CES1 expression and activity were found in neonates relative to adults and increased gradually with age [9,10]. Conversely, modeling geriatric populations introduces different physiological complexities. Although hepatic CES1 expression remains stable during healthy aging [9], geriatric pharmacokinetics are confounded by systemic senescence. Aging is associated with decreased organ blood flow, declining glomerular filtration rate (GFR), and altered body composition [11]. Given that the activation and clearance of high-affinity ester prodrugs are perfusion-limited, whereas the elimination of their active metabolites is renally driven, age-related organ involution can significantly perturb this coupled activation-elimination kinetics. However, it is impractical and cost-prohibitive to conduct human pharmacokinetic studies in pediatric and geriatric populations of all drugs metabolized by CES1 to inform dose adjustment. Although several PBPK frameworks have successfully characterized CES1 substrates, such as methylphenidate, spironolactone, or oseltamivir [10,12,13,14,15,16], their applicability across pediatric and geriatric populations remains unexploited. Therefore, it is necessary to develop an integrated, mechanistic PBPK model that couples age-dependent CES1 ontogeny with senescent physiological decline to simultaneously predict prodrug–metabolite exposure.

The present study focuses on three widely used CES1-activated prodrugs: oseltamivir, enalapril, and cilazapril. Oseltamivir is approved for the treatment and prophylaxis of influenza and serves as an ideal model drug due to its widespread clinical use and abundant pharmacokinetic data. Enalapril and cilazapril, two commonly prescribed ACEIs, are used for the management of hypertension and heart failure. All three agents undergo CES1-mediated hydrolysis to generate their respective active metabolites: oseltamivir carboxylate (OSC), enalaprilat, and cilazaprilat. This study aims to establish a mechanism-driven foundation for evaluating the pharmacokinetic behavior of CES1-activated prodrugs across pediatric and geriatric cohorts, thereby providing a rational modeling template to characterize age-dependent exposure variability in these vulnerable populations.

## 2. Materials and Methods

### 2.1. Data Collection

Specific selection criteria were established for the development of the CES1-mediated prodrug–metabolite PBPK model. First, candidate prodrugs were required to be primarily activated by CES1 without significant alternative metabolic pathways. Second, it was imperative that plasma concentration–time profiles were available in adults and pediatric/geriatric populations. Studies were excluded if they involved subjects with severe hepatic or renal impairment, intestinal disease, or heart failure, which could confound the assessment of age-related PK alterations. Based on these criteria, oseltamivir, enalapril, and cilazapril met the requirements and were included in the present study as clinically relevant CES1-activated prodrugs with distinct pharmacokinetic characteristics. Pharmacokinetic data were systematically retrieved from PubMed and Google Scholar using Boolean operators: (‘oseltamivir’ OR ‘enalapril’ OR ‘cilazapril’) AND ‘pharmacokinetics’. Published concentration–time profiles were digitized using Engauge Digitizer (version 12.1).

### 2.2. Development and Validation of the Prodrug–Metabolite PBPK Model

A dynamic, age-dependent whole-body PBPK model was constructed to simultaneously characterize the pharmacokinetics of both CES1-activated prodrugs and their active metabolites (Figure 1A). The framework was structured with two parallel sets of 13 anatomical compartments, representing the prodrug and the active metabolite, respectively. These two sub-models were interconnected via the hepatic compartment, where the CES1-mediated bioactivation of the prodrug was assumed to occur exclusively. Although extrahepatic CES1 expression (e.g., in the lung and brain) exists, CES1 expression is overwhelmingly enriched in the liver relative to extrahepatic tissues [7,8]. Each system integrated compartments for the arterial and venous blood, lungs, heart, brain, muscle, adipose tissue, skin, spleen, and the rest of the body, with the liver and kidneys defined as the primary organs responsible for drug elimination. The gut was segmented into the stomach, duodenum, jejunum, ileum, cecum, and colon, with each segment further divided into the intestinal wall and lumen. The model was parameterized using drug-specific and system-specific data. Mass balance differential equations (detailed in the Supplementary Information) were implemented and solved using the Phoenix NLME platform (Pharsight, St. Louis, MO, USA).

Monte Carlo simulations were performed for each scenario with 1000 virtual subjects. To capture the impact of parameter uncertainty on population simulations, variability was introduced for several key parameters, including the intestinal effective permeability coefficient ($P_{eff}$), the free fraction of the drug in blood ($f_{ub}$), hepatic intrinsic clearance (${CL}_{int,CES1}$), and renal intrinsic clearance (${CL}_{int,r}$). A 20% coefficient of variation (CV) was assumed for these parameters in the absence of reliable population-specific variability estimates, following the approach used in previous PBPK studies [16,17,18]. The impact of different CV assumptions was further evaluated through sensitivity analysis.

The PBPK model was established using a sequential development and validation workflow (Figure 1B). Clinical PK datasets from adults [19,20,21] were used for model development to determine appropriate distribution models and refine drug-specific parameters. Additional cohorts from the model development studies were used for internal validation, whereas independent adult clinical datasets were reserved for external validation of the adult PBPK model. Following successful evaluation in a virtual population of 1000 healthy adults, the framework was adapted to pediatric and geriatric populations by updating system-specific physiological and biochemical parameters, together with relevant drug-specific parameters. Pediatric and geriatric clinical datasets were used exclusively for external validation of the age-adapted model, without model refitting based on clinical observations. The validated framework was subsequently applied to predict systemic PK profiles of both prodrugs and their active metabolites in 1000 virtual individuals across different age groups, and predictions were compared with published clinical observations.

### 2.3. Physiological Parameters

#### 2.3.1. Organ Mass, Blood Flow Rate, Body Weight (BW), and Height

Age-specific organ mass and blood flow rates for pediatric and adult populations were primarily sourced from Chang et al. [22], with cardiac and adipose blood flow rates supplemented from Björkman et al. [23]. The equations describing the relationship between organ mass/blood flow rate and age are summarized in Tables S1–S3. Anthropometric correlations (body weight and height versus age) for individuals aged 0–20 years are presented in Table S4. For adults, it was assumed that physiological parameters were stable, and values from 19-year-old subjects were adopted as representative (Table 1).

For geriatric populations, physiological parameters, including organ mass and blood flow, were primarily sourced from Schlender et al. [24]. Individuals aged 60, 70, and 80 years were used to represent geriatric populations. The organ mass of the stomach and intestines was scaled from adult data using the body weight ratio. Blood flow rates for the stomach, intestines, and spleen were adjusted according to the total splanchnic blood flow rates [24], using proportions corresponding to those for adults. Blood volume in elderly individuals ($V_{bl,\;elderly}$) was calculated using the formula [25]:

(1) $$V_{bl,elderly}=e^{\left(0.067\times \mathrm{BSA}-0.0025\times \mathrm{age}+1.7\right)}$$

where body surface area (BSA, $m^{2}$) can be obtained using the formula [26]:

(2) $$BSA\;=0.0235\times H^{0.42246}\times {BW}^{0.51456}$$

where H is the height (cm), and BW is the body weight (kg).

#### 2.3.2. Gastrointestinal Radii and Transit Rates

For pediatric and adult populations, the radii (r,m) of the duodenum, jejunum, and ileum were calculated using the formula [27]:

(3) $$r=\frac{0.016\times BSA+0.0159}{2}$$

where BSA can be calculated using Equation (2).

The gastric emptying rate ($K_{t0}$) for adults was set at 0.0462 (min^−1^) [28], and intestinal transit rates ($K_{t1}$, $K_{t2}$, $K_{t3}$, $K_{t4}$, and $K_{t5}$) were set at 0.07, 0.03, 0.04, 0.003, and 0.001 (min^−1^) [29], respectively. According to the review by Kaye et al. [30], the gastric emptying times for different pediatric age groups were as follows: 68 min (54–82 min) for neonates (0–27 days), 41 min (12–70 min) for infants (1–23 months), 41 min (12–70 min) for children (2–11 years), and 75 min (12–138 min) for adolescents (12–18 years). Intestinal transit rates in pediatric populations were nearly identical to those observed in adults throughout development. For geriatric populations, intestinal radii were assumed to be equivalent to those of adults. The gastric emptying rate was approximately half of the adult value (resulting in a prolonged gastric emptying time), whereas the small intestinal transit time was kept constant and comparable to adults, consistent with established clinical observations [31,32].

### 2.4. Physicochemical and Pharmacokinetic Parameters

#### 2.4.1. Adult Physicochemical and Pharmacokinetic Parameters

Drug-specific input parameters used in the adult PBPK model are summarized in Table 2. The well-stirred, perfusion-rate-limited model was used to model distribution into tissues, with partition coefficient ($K_{t:p}$) values estimated using published tissue composition-based equations [33].

#### 2.4.2. Scaling to Pediatric Populations

In pediatric PBPK modeling, age-related variations in ${CL}_{int,h}$, ${CL}_{int,r}$, and $f_{up}$ should be considered. All the tested drugs are mainly bound to plasma albumin (ALB). ALB levels are lower in newborns than in adults and gradually increase with age. Accordingly, the $f_{up}$ of the drug decreases with age. Pediatric $f_{up}$ ($f_{up,child}$) can be calculated using the following equation [45]:

(4) $$f_{up,child}=\frac{1}{1+\frac{{ALB}_{child}\times \left(1-f_{up,adult}\right)}{{ALB}_{adult}\times f_{up,adult}}}$$

where the pediatric plasma albumin concentration (${ALB}_{child}$, g/L) is calculated using the following formula [27,46,47]:

(5) $${ALB}_{child}=1.1287\times \ln\left(Age\right)+33.746$$

where age is in years.

Hepatic intrinsic clearance for children (${Cl}_{int,h,child}$) is calculated as follows:

(6) $${CL}_{int,h,child}=\frac{{CL}_{int,h,adult}\times R_{CES1,child}\times W_{h,child}}{W_{h,adult}}$$

where $W_{h}$ represents liver weight. It is assumed that the intrinsic catalytic efficiency of CES1 is constant, and age-related differences in CES1-mediated metabolism are driven by changes in hepatic CES1 protein abundance. The CES1 expressions in pediatric livers relative to adults ($R_{CES1,child}$) are described by [48]:

(7) $$R_{CES1,child}=0.1491\times \ln\left(Age\right)+0.5704$$

where age is in years.

For renal clearance, the pediatric ${CL}_{r}$ (${CL}_{r,child}$) is calculated using the following equation [23]:

(8) $${CL}_{r,child}=\;({GFR}_{child}\times f_{up,child})/({GFR}_{adult}\times f_{up,adult})\;\times \;{CL}_{r,adult}$$

where the GFR in pediatrics is related to BW and age [49]:

(9) $$GFR=2.6\times {BW}^{0.662}\times e^{-0.0822\times age}+8.14\times {BW}^{0.662}\times \left(1-e^{-0.0822\times age}\right)$$

Here, the ${CL}_{r,adult}$ was derived from clinical total renal clearance, thereby capturing net renal elimination (combining both glomerular filtration and active tubular secretion). For pediatric extrapolation, this net clearance was scaled using the ratio of ($f_{up}$ × GFR) as a functional surrogate for overall renal maturation. Subsequently, ${CL}_{int,r,child}$ can be back-calculated by using Equation (S12).

#### 2.4.3. Scaling to Geriatric Populations

The unbound drug fraction in the elderly ($f_{up,elderly}$) was estimated using Equation (4), where ALB in the elderly (${ALB}_{elderly}$) is described by the following equation [47]:

(10) $${ALB}_{elderly}=51.4-0.107\times Age$$

In the elderly, CES1 expression is comparable with that in adults; therefore, the expression levels of CES1 in geriatric liver relative to adults ($R_{CES1,elderly}$) were assumed to be 1. Hepatic intrinsic clearance for geriatrics (${Cl}_{int,h,elderly}$) was estimated using Equation (6).

For renal clearance, this parameter was adjusted for the elderly as follows:

(11) $${CL}_{r,elderly}=\frac{{GFR}_{elderly}\times f_{up,elderly}}{{GFR}_{adult}\times f_{up,adult}}\times {CL}_{r,\;adult}$$

where GFR declines by about 0.75 mL/min per year with aging in healthy subjects [50]. The ratio of $GFR\times f_{up}$ represents overall functional nephron decay. Physiologically, age-related renal decline reflects a global loss of functional nephrons, causing filtration and active secretion to decline in parallel. ${CL}_{int,r,elderly}$ can be back-calculated by using Equation (S12).

### 2.5. Sensitivity Analyses

To assess the impact of model parameters on systemic exposure, a local sensitivity analysis was performed. For oseltamivir/OSC, sensitivity analyses were conducted in three representative populations: adults receiving a 75 mg oral dose, pediatric subjects aged 1 month receiving a 2 mg/kg oral dose, and geriatric subjects aged 80 years receiving a 75 mg oral dose. Seven key parameters were investigated, including four physiological parameters ($K_{t0}$, $Q_{HA}$,$Q_{r}$, and GFR) and three drug-specific parameters ($P_{eff}$, $f_{ub}$, and ${CL}_{int,h}$). $K_{t0}$, $Q_{HA}$,$Q_{r}$, and $P_{eff}$ were varied by a factor of 1/3 to 3, while the GFR and $f_{ub}$ of oseltamivir were varied by a factor of 1/3 to 1.5. Additionally, the $f_{ub}$ of OSC was varied by a factor of 1/3, and ${CL}_{int,h}$ was varied by a factor of 1/10 to 10. In addition, an additional sensitivity analysis was conducted to evaluate the impact of variability assumptions on population simulations. The CV of the key parameters was varied from 10% to 30% (10%, 20%, and 30%). For enalapril/enalaprilat and cilazapril/cilazaprilat, sensitivity analyses were performed in adult populations following oral administration at clinically relevant doses.

### 2.6. Analysis of Model Predictability

Model predictive performance was evaluated using qualitative and quantitative approaches. First, the predicted and observed concentration–time curve profiles were visually compared. Second, predictive accuracy was assessed based on whether the predicted PK parameters, specifically area under the curve (${AUC}_{0-t}$) and maximum plasma concentration ($C_{max}$), fell within a 0.5- to 2.0-fold range of the corresponding observed values [16,17,28,29]. Third, systemic bias and precision were quantified using the average fold error (AFE), average absolute prediction error (PE%), and geometric mean fold error (GMFE). Model predictability was categorized based on the following criteria: AFE values of 0.8–1.25 were defined as satisfactory, 0.5–0.8 or 1.25–2.0 as acceptable, and <0.5 or >2.0 as poor; PE% values of ≤25% were defined as satisfactory, 25–50% as acceptable, and >50% as poor; and GMFE values of ≤1.25 were defined as satisfactory, 1.25–2.0 as acceptable, and >2.0 as poor [17]. These statistical metrics were calculated as follows:

(12) $$AFE=10^{\frac{1}{n}\sum log\frac{Predi}{Obsi}}$$

(13) $$PE\%=Geomean(\left|\frac{Predi-Obsi}{Obsi}\right|)\times 100$$

(14) $$GMFE=10^{\frac{\sum \left|log(\frac{Predi}{Obsi})\right|}{n}}$$

## 3. Results

### 3.1. Dataset Overview

A total of 25 clinical studies were collected to assess the pharmacokinetics of oseltamivir, cilazapril, and enalapril, as well as their active metabolites (Table S6). Data on all three drugs were available for adults. Oseltamivir was evaluated in five studies covering both oral administration (75–150 mg) and intravenous administration (100–400 mg); cilazapril was assessed in five studies with oral doses ranging from 0.5 to 5 mg; and enalapril was examined in eight trials with oral doses of 10 to 20 mg.

Pediatric data were available for oseltamivir and enalapril. The pharmacokinetics of oseltamivir were described in four studies covering neonates, infants, and children following oral administration. Enalapril data came from a single study providing age-stratified results from infants to adolescents. All three drugs were investigated in geriatric populations. Oseltamivir was included in two studies, one of which enrolled the elderly with a mean age of 84 years. One study each for cilazapril and enalapril included the elderly, with mean ages of around 70 years.

### 3.2. PBPK Model Development and Validation for Adults

The whole-body prodrug–metabolite PBPK model was initially established using adult clinical PK data following oral administration of 75 mg oseltamivir [19], 10 mg enalapril [20], and 2.5 mg cilazapril [21]. Model development datasets were utilized to refine structural configurations and input model parameters. Using the finalized adult parameters (Table 1 and Table 2), plasma concentration–time profiles for both prodrugs and their active metabolites were predicted simultaneously. Studies designated for model development are marked with asterisks in Figure 2A,F,H. Detailed trial characteristics and dosing regimens are summarized in Table S6. For oseltamivir and OSC, data from the 75 mg non-obese cohort of Jittamala et al. (2014) [19] were used for model development, whereas the remaining cohort within the same study served as the internal validation. The remaining independent studies were held out for external validation. The majority of observed plasma concentration data points fell well within the predicted 5th–95th prediction intervals across all evaluation cohorts (Figure 2A–E). Similarly, for enalapril and enalaprilat, data from Ohnishi et al. (1989) [20] were used for model development, with six external studies reserved for independent validation. The model successfully captured the coupled kinetics of enalapril bioactivation and enalaprilat elimination across all datasets (Figure 2F,G). For cilazapril and cilazaprilat, data from Williams et al. (1989) [21] were utilized for model development, while four independent studies were used for external validation. The predicted concentration–time profiles for both cilazapril and cilazaprilat demonstrated high concordance with clinical observations (Figure 2H–K).

Quantitative predictive performance was evaluated by comparing calculated PK parameters, including the AUC and $C_{max}$, against observed values. Overall, 97.96% (48/49) of predicted AUC values and 100% (49/49) of predicted $C_{max}$ values fell within the 2-fold model acceptance criteria (Figure 3, dashed lines). In addition, 89.80% (44/49) of the predicted AUC values and 93.88% (46/49) of the $C_{max}$ values were within a more stringent 1.5-fold range of the observed data (Figure 3, dotted lines). The calculated AFE, PE%, and GMFE values for both $C_{max}$ and AUC indicated that all predictions for adults were within satisfactory or acceptable ranges (Table 3). Collectively, these data indicated that the adult CES1-mediated prodrug–metabolite PBPK model was built successfully.

### 3.3. Prediction of the Age-Related Impact on Pharmacokinetics

After verifying the predictive performance of the CES1-mediated prodrug–metabolite PBPK model in adults, the framework was extrapolated to pediatric and geriatric populations by integrating age-dependent physiological and PK parameters (Figure 4 and Figure 5).

For oseltamivir, which possessed the largest and most comprehensive clinical dataset, the model successfully captured the longitudinal PK profiles in pediatric and geriatric populations, with the majority of the observed data falling within the 5th and 95th percentiles of the simulated population (Figure 4 and Figure 5). Although the model overestimated oseltamivir concentrations in neonates, the predicted exposure for OSC remained highly consistent with clinical observations (Figure 4A,B). Thus, the model showed potential for predicting the PK profiles of oseltamivir and OSC across a broad range of ages. To further evaluate the impact of age within the geriatric population, an age-stratified simulation was performed for oseltamivir. The model predicted a progressive, age-dependent increase in systemic exposure, yielding predicted OSC AUC ratios of 1.29, 1.59, and 1.89 for subjects aged 60, 70, and 80 years, respectively, relative to adults (Figure S1). For enalapril, supported by a moderate sample size of clinical studies spanning four distinct pediatric age cohorts (2 months to 15 years) and one geriatric cohort (70 years), the model showed reasonable agreement with the observed concentration–time profiles for both the prodrug and enalaprilat (Figure 4O–R and Figure 5E). For cilazapril, clinical validation was limited by the smallest sample size, with observed data available only for a single geriatric cohort (70 years). The model successfully predicted the systemic exposure of its active metabolite, cilazaprilat (Figure 5F). Therefore, cilazapril should be considered a preliminary proof-of-concept extension of the framework, particularly for geriatric application, rather than comprehensive validation across age groups.

Quantitative evaluation of the model‘s performance in pediatric and geriatric populations is summarized in Figure 6. In the pediatric population, 96.67% (29/30) of the predicted AUC values and 86.67% (26/30) of the $C_{max}$ values fell within a 2-fold range of the observed data. In the geriatric population, all predicted AUC and $C_{max}$ values were within the 2-fold acceptance criteria. Furthermore, 80.00% (24/30) of AUC and 50% (15/30) of $C_{max}$ for the pediatric population, as well as 66.67% (8/12) of AUC and 66.67% (8/12) of $C_{max}$ for the geriatric population, were within a 1.5-fold range of the observed values. The AFE, PE, and GMFE values were calculated using the parameters $C_{max}$ and ${AUC}_{0-t}$. The results showed that the predictions for pediatrics and geriatrics were within acceptable ranges (Table 4). These results support the utility of the PBPK framework for evaluating age-related changes in pharmacokinetics across diverse populations. However, the model showed a tendency to overpredict OSC Cmax in some pediatric cohorts. This discrepancy may be partly attributable to the relatively sparse sampling schedules of pediatric clinical PK studies, as well as the fact that the available pediatric datasets were primarily derived from patient populations rather than healthy children. For geriatric populations, the predicted PK parameters based on the currently available clinical datasets generally remained within the predefined acceptable range, although further validation using additional well-characterized clinical datasets is warranted.

### 3.4. Sensitivity Analyses

A local sensitivity analysis was performed to quantify the impact of seven physiological and drug-specific parameters on the PK profiles of the evaluated CES1-activated prodrugs and their active metabolites. Sensitivity analyses were conducted for oseltamivir/OSC, enalapril/enalaprilat, and cilazapril/cilazaprilat following oral administration of clinically relevant adult doses (oseltamivir 75 mg, enalapril 10 mg, and cilazapril 2.5 mg).

For oseltamivir and OSC, the sensitivity landscapes conducted were highly consistent across the three age groups evaluated (adults, 1-month-old infants, and 80-year-old subjects), exhibiting identical trends in the parameters’ impact (Figure 7 and Figures S2 and S3). For prodrug oseltamivir, ${CL}_{int,h}$ was identified as the most influential determinant, showing a strong negative correlation with systemic exposure. For oseltamivir, $f_{ub}$, $P_{eff}$, and $K_{t0}$ showed moderate effects. Specifically, reducing $K_{t0}$ to one-third of its baseline resulted in delayed absorption and a lower peak concentration, whereas a 3-fold increase in $K_{t0}$ led to an earlier and higher peak concentration. Conversely, alterations in $Q_{HA}$, $Q_{r}$ and GFR had negligible effects on oseltamivir profiles. For the active metabolite OSC, the sensitivity profile differed. The primary determinant was its $f_{ub}$, followed by GFR, $Q_{r}$, and $P_{eff}$. While a reduction in ${CL}_{int,h}$ predictably reduced OSC exposure, an increase in ${CL}_{int,h}$ resulted in a plateau effect, with only marginal additional gains in exposure. Notably, a 10-fold reduction in ${CL}_{int,h}$ led to a 7-fold increase in oseltamivir exposure, whereas the systemic exposure of OSC was decreased by only 27.6% (Figure 7G,N). Moreover, OSC exposure was found to be insensitive to variations in the $f_{ub}$ of oseltamivir and $Q_{HA}$.

Additional sensitivity analyses of enalapril/enalaprilat and cilazapril/cilazaprilat revealed generally similar parameter–exposure relationships to those observed for oseltamivir/OSC, although differences were observed for some individual parameters (Figures S4 and S5). Especially, variations in $P_{eff}$ exerted a relatively smaller influence on the systemic exposure of cilazapril and cilazaprilat (Figure S5D,K) compared to oseltamivir/OSC and enalapril/enalaprilat (Figure 7D,K and Figure S4D,K). In addition, increased CLint,h had only a modest effect on OSC exposure (Figure 7N), whereas it markedly affected the exposure of enalaprilat and cilazaprilat (Figures S4N and S5N).

Additionally, an uncertainty analysis was performed using oseltamivir/OSC as a representative case. Sensitivity analyses using CV values ranging from 10% to 30% demonstrated that increased variability assumptions resulted in wider prediction intervals, whereas the predicted mean concentration–time profiles remained comparable across different CV settings (Figure S6).

## 4. Discussion

This study developed a whole-body PBPK framework to characterize the disposition of three CES1-activated prodrugs and their active metabolites across different age groups. The model demonstrated adequate predictive performance, with more than 80% of predicted Cmax and AUC values within 2-fold of the corresponding observed values. Furthermore, AFE, PE, and GMFE values indicated that the model predictions were within acceptable limits of precision and bias across most cohorts. Oseltamivir and enalapril were evaluated across pediatric, adult, and geriatric populations, whereas cilazapril was assessed in adult and geriatric populations due to the limited availability of pediatric clinical data and served as an extension case to further examine the applicability of the framework to additional CES1-activated prodrugs. Collectively, these findings support the utility of this framework for evaluating age-related PK changes associated with CES1-mediated prodrug activation.

To characterize the clinical impact of CES1 variability, several PBPK models have been developed. PBPK frameworks for methylphenidate have integrated the influences of pharmacogenetics and drug–drug interactions on drug exposure [12,13]. For oseltamivir, prior modeling efforts evaluated the kinetic consequences of impaired CES1 activity induced by ethanol co-administration or genetic loss-of-function polymorphisms [14]. In terms of specific populations, Boberg et al. [10] utilized age-dependent absolute protein abundance to predict oseltamivir disposition in infants, while Soliman et al. [15] applied model-informed strategies to optimize spironolactone dosing in neonates and infants. Recently, a semi-PBPK model was developed to simultaneously simulate both CES1-metabolized drugs and their active metabolites in liver cirrhosis [16]. Expanding upon these findings, our study advances this modeling strategy by establishing a whole-body PBPK framework. Compared with previous semi-PBPK approaches [16], this whole-body framework incorporates system-wide physiological parameters and enzymatic ontogeny functions. This allows for mechanistic extrapolation of prodrug activation and active metabolite disposition from pediatrics to geriatrics. Collectively, existing frameworks remain constrained to single compounds or specific pathologies. Therefore, establishing a generalized, whole-body PBPK platform that captures these coupled prodrug–metabolite biotransformation kinetics across both pediatric and geriatric populations bridges a critical knowledge gap. Our PBPK framework provided reliable exposure predictions across age groups with limited empirical adjustment, supporting its mechanistic basis for extrapolating drug disposition across populations. Most drug-specific parameters were derived from in vitro, in vivo, or literature-supported physiological relationships. A limited number of parameters without direct experimental or literature support were estimated using clinical PK data during model development. Specifically, the Peff value of cilazapril was fitted using adult clinical PK data. By simultaneously modeling both the prodrug and its active metabolite, the framework successfully captures the coupled dynamics of bioactivation and clearance, offering a scalable template for other CES1-activated agents to inform exposure risk assessments and trial design in data-limited populations.

Overall, extrapolation of the PBPK framework to the pediatric population demonstrated adequate predictive performance, with the majority of predicted PK parameters falling within the 2-fold error boundary. A notable divergence, however, occurred within the neonatal cohort under one month of age, where the simulations revealed a systemic overprediction of oseltamivir exposure, while predictions for OSC remained highly consistent with observed clinical data. This discrepancy may reflect additional physiological factors that are not explicitly represented in the current gastrointestinal absorption module. The current framework incorporates key developmental processes, including age-dependent changes in CES1 ontogeny, plasma protein binding, renal maturation, and intestinal permeability. However, due to the lack of reported quantitative physiological data describing intestinal pH profiles in neonates [30], age-related variations in intestinal pH were not explicitly incorporated. Nevertheless, neonatal gastrointestinal physiology differs substantially from that of older infants, including higher variability in gastric pH and frequent milk feeding, which may influence luminal drug dissolution, stability, and absorption processes. Therefore, differences in gastrointestinal pH and related physiological conditions may represent potential contributors to the observed neonatal discrepancy. In the geriatric population, the model predicted a significant increase in OSC and enalaprilat exposure following oral administration compared to adults, suggesting a higher risk of dose-related adverse effects. Interestingly, the model predicted minimal changes in cilazaprilat exposure, reflecting drug-specific sensitivity to physiological aging. This divergence may be attributed to the complex interplay between declining renal function and age-related alterations in plasma protein binding.

Local sensitivity analysis further elucidated the core drivers governing prodrug–metabolite disposition. Taking oseltamivir as a representative case, systemic exposure of the prodrug across pediatric, adult, and geriatric populations was primarily influenced by hepatic CES1 activity and intestinal effective permeability, confirming that prodrug systemic exposure is strictly an absorption- and metabolism-limited process. However, the variation in the systemic exposure of the active metabolite, OSC, was far less pronounced than that observed for oseltamivir in response to alterations in CES1 activity. Mechanistically, this uncoupled responsiveness is attributable to the fact that hydrolysis into OSC represents the prodrug’s sole metabolic pathway; even under conditions of compromised CES1 activity, the resulting prolongation of prodrug residence time ensures that oseltamivir is eventually biotransformed. Conversely, the active therapeutic metabolites were profoundly sensitive to their own unbound fraction, renal blood flow, and GFR. Importantly, this sensitivity pattern suggests that the age-related decline in renal function will exacerbate systemic exposure to OSC, significantly heightening the risk of accumulating active metabolites. This provides a physiological rationale for understanding age-dependent PK variations, highlighting key drivers to inform exposure risk assessment in vulnerable populations.

Several limitations require acknowledgment. First, some limitations are inherent to the current PBPK framework and its underlying assumptions. For age extrapolation, total renal clearance was assumed to scale proportionally with $f_{up}$ × GFR. This reflects the current lack of reliable quantitative data regarding the specific developmental ontogeny and age-related decay profiles of the renal transporters involved (i.e., OAT3 and MRPs). Additionally, the model assumes constant CES1 catalytic efficiency per unit enzyme abundance across age groups. It may not fully account for substrate-specific differences in CES1 kinetic properties, which could introduce uncertainties when extrapolating the model to other CES1 substrates. Moreover, the structural simplification of the absorption compartment, without accounting for pH differences, may introduce prediction variances for neonates. Furthermore, adult physiological parameters in the present framework were established using a 19-year-old reference population, representing a mature young-adult physiological state. Although most physiological determinants of drug disposition remain relatively stable during early adulthood, age-related changes in renal function occur in older adults (>40 years). Therefore, applying a young-adult reference physiology to older adult populations may introduce a potential bias toward overestimation of renal clearance capacity. Second, the representation of inter-individual variability within virtual populations remains limited. In the framework, variability was incorporated through predefined distributions for selected model parameters. Although age-dependent physiological changes were considered, population distributions of some physiological characteristics, including body weight, organ size, and blood flow rates, were not explicitly modeled. Therefore, the simulated 5th–95th percentile intervals reflect the variability captured by the implemented parameter distributions rather than the full spectrum of population variability. Third, limitations are also associated with the availability and characteristics of clinical datasets used for model validation. Clinical PK data in special populations remain limited. Pediatric PK studies are predominantly conducted in patients rather than healthy children because of ethical considerations. Although studies involving severe hepatic or renal dysfunction were excluded to minimize potential confounding, disease-related heterogeneity and limited sampling may still contribute to variability in observed PK profiles. In addition, available geriatric PK datasets for model validation remain relatively limited. Although current predictions remained within the acceptance criterion, further validation with larger and better-characterized clinical datasets is warranted to further confirm model robustness in elderly populations. In conclusion, this whole-body prodrug–metabolite PBPK model serves as a valuable platform for CES1-activated prodrugs. Future iterations should integrate disease-state physiology and expanded datasets from vulnerable populations to further enhance clinical applicability. Coupling this framework with PD endpoints will ultimately support model-informed dose optimization in real-world clinical settings.

## 5. Conclusions

This study establishes an integrated whole-body PBPK framework tailored for evaluating CES1-mediated prodrug–metabolite disposition. By integrating age-specific parameters, the model successfully predicts the pharmacokinetics of oseltamivir, cilazapril, and enalapril across diverse age groups. It provides a mechanistic approach for predicting the pharmacokinetics of CES1-activated prodrugs, particularly in populations where clinical data are limited or difficult to obtain.

## Figures and Tables

**Figure 1:**
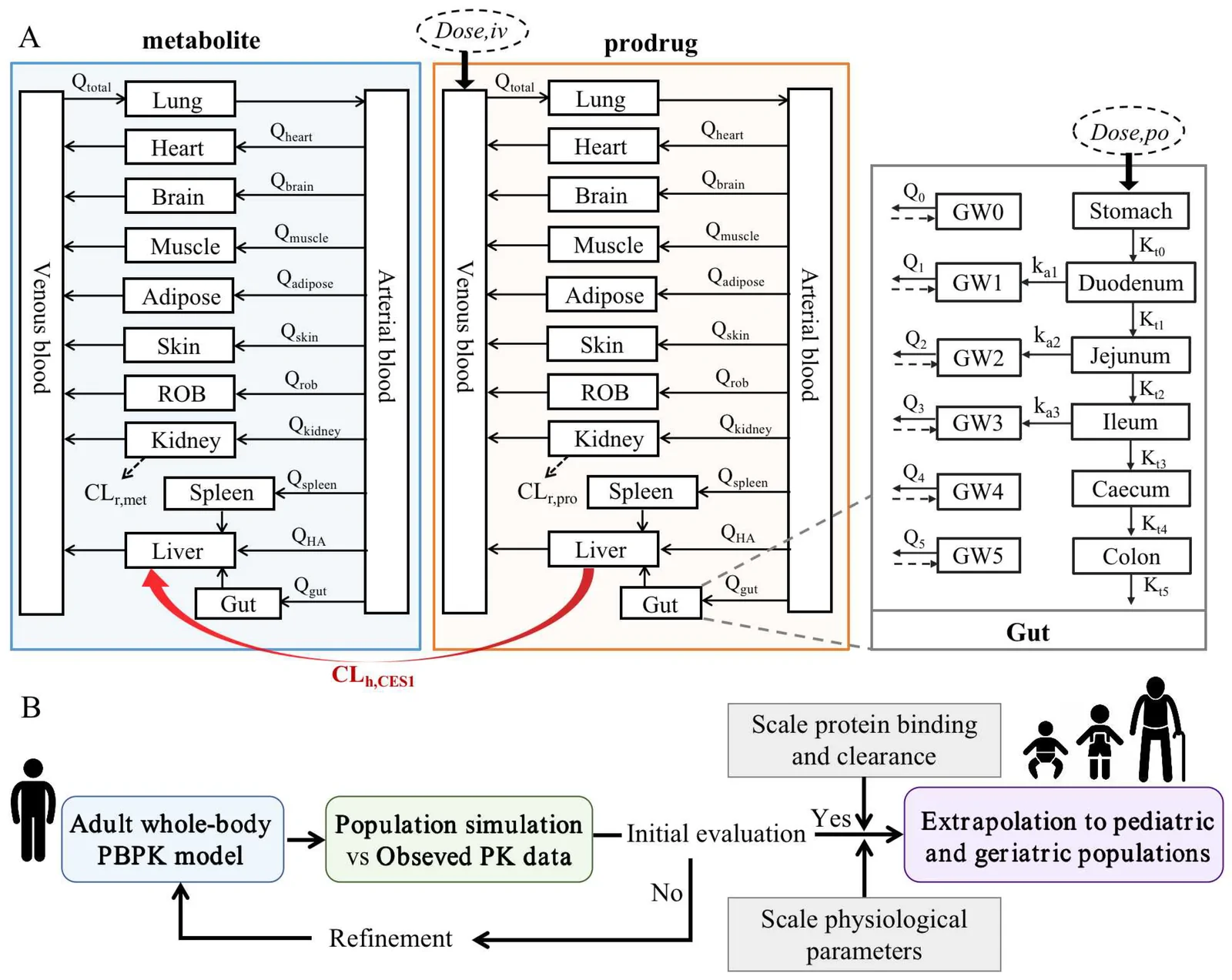
Schematic diagram of the PBPK model. (**A**) Structure of the CES1-mediated prodrug–metabolite PBPK model. (**B**) Workflow for development and validation of the CES1-mediated PBPK prodrug–metabolite model across the human lifespan. ROB: rest of body; GW: gut wall.

**Figure 2:**
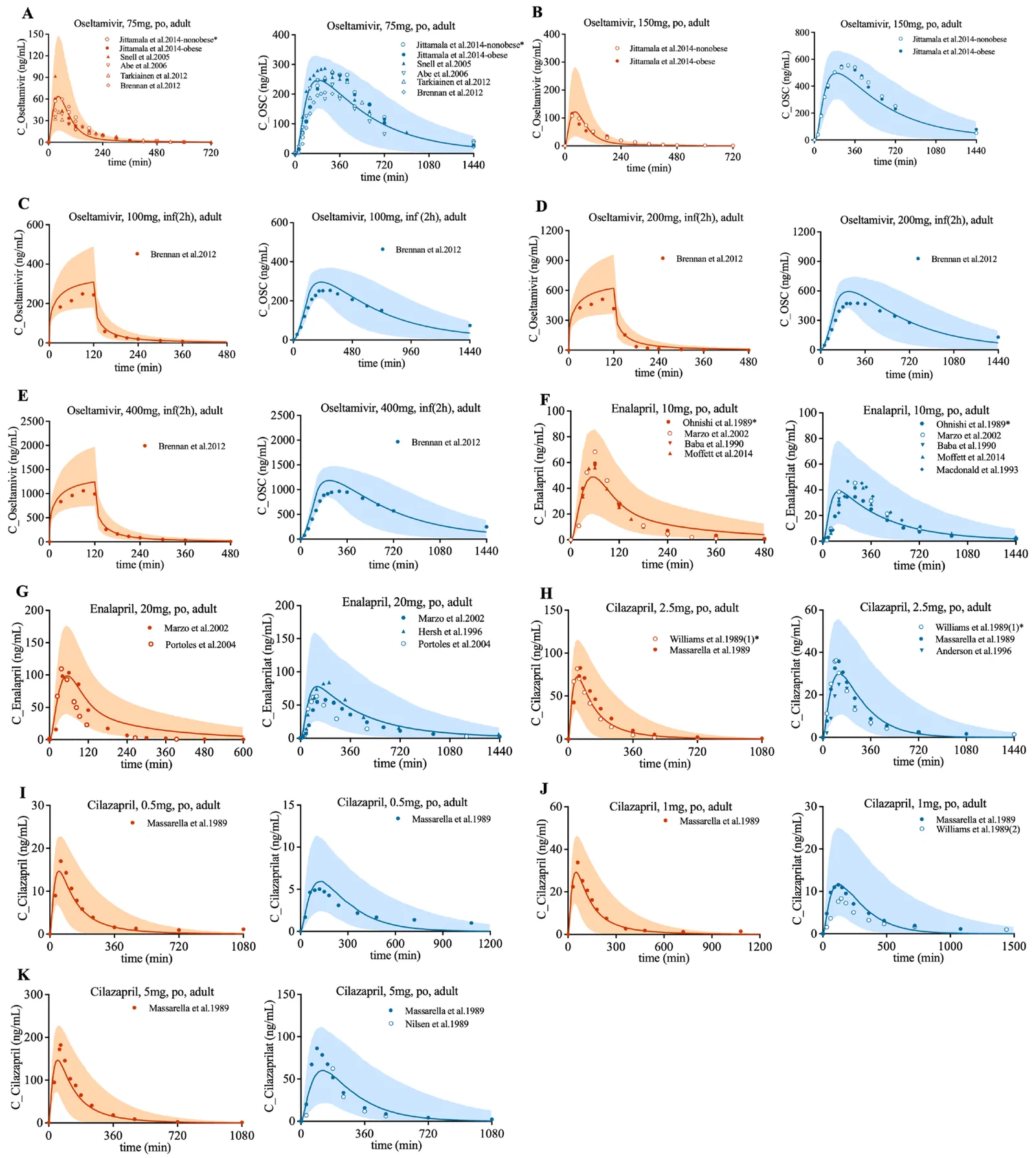
Plasma concentration–time profiles of oseltamivir, cilazapril, enalapril (orange), and their active metabolites (blue) in adults. (**A**) Oral 75 mg oseltamivir [19,51,52,53,54]; (**B**) oral 150 mg oseltamivir [19]; (**C**) intravenous infusion of 100 mg oseltamivir over 2 h [51]; (**D**) intravenous infusion of 200 mg oseltamivir over 2 h [51]; (**E**) intravenous infusion of 400 mg oseltamivir over 2 h [51]; (**F**) oral 10 mg enalapril [20,55,56,57,58]; (**G**) oral 20 mg enalapril [56,59,60]; (**H**) oral 2.5 mg cilazapril [21,61,62]; (**I**) oral 0.5 mg cilazapril [61]; (**J**) oral 1 mg cilazapril [43,61]; (**K**) oral 5 mg cilazapril [61,63]. The solid lines indicate the mean predicted concentrations, and the shaded regions correspond to the 5th–95th percentile ranges. The asterisk (*) denotes cohorts used for model development.

**Figure 3:**
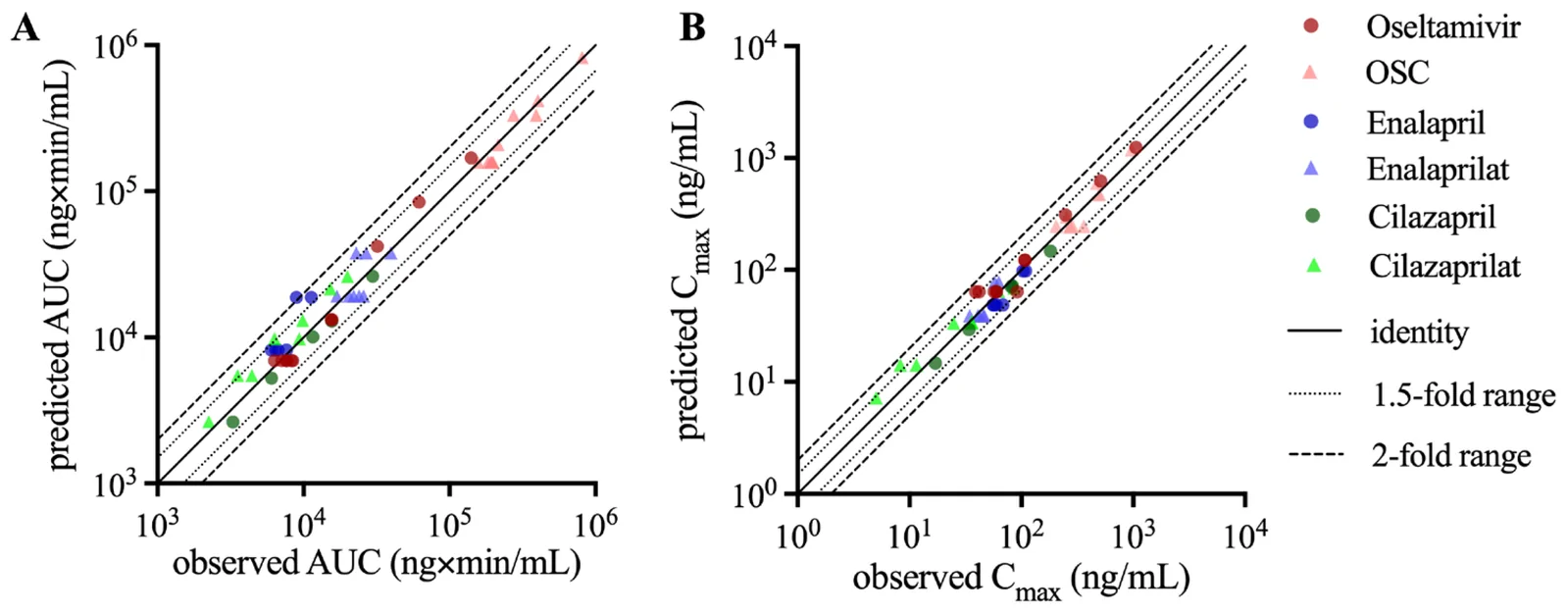
Goodness-of-fit plots showing predicted versus observed (**A**) AUC and (**B**) $C_{max}$ in adults. The solid line represents the line of identity, and the dotted and dashed lines indicate the 1.5-fold and 2-fold error ranges. AUC: area under the plasma concentration–time curve; $C_{max}$: maximum plasma concentration.

**Figure 4:**
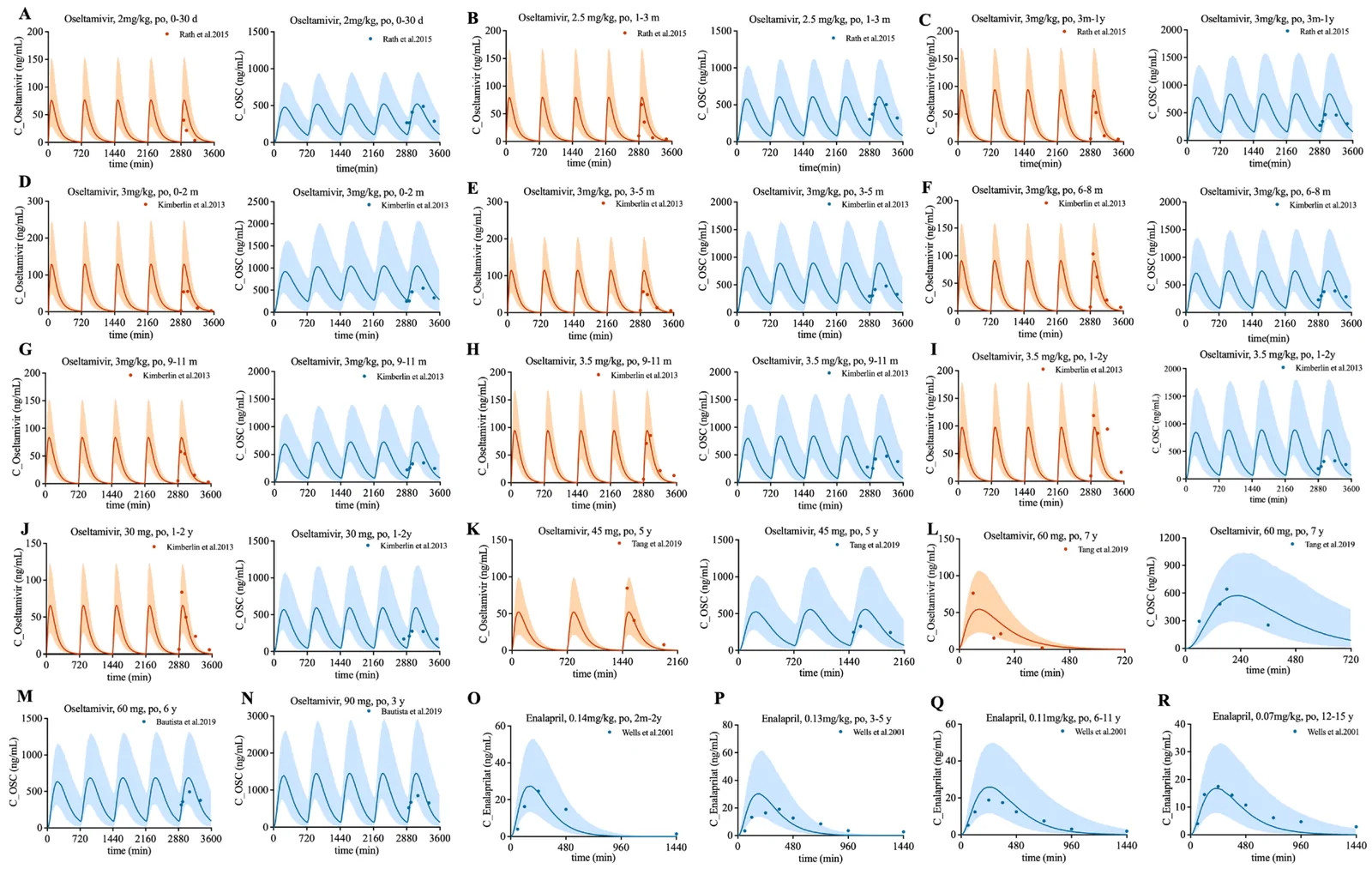
Plasma concentration–time profiles of oseltamivir, enalapril (orange), and their active metabolites (blue) in the pediatric population. (**A**) Oral 2 mg/kg oseltamivir in children aged 0–30 days [64]. (**B**) Oral 2.5 mg/kg oseltamivir in children aged 1–3 months [64]. Oral 3 mg/kg oseltamivir in children aged (**C**) 3–12 months [64], (**D**) 0–2 months [65], (**E**) 3–5 months [65], (**F**) 6–8 months [65], and (**G**) 9–11 months [65]. Oral 3.5 mg/kg oseltamivir in children aged (**H**) 9–11 months and (**I**) 1–2 years [65]. (**J**) Oral 30 mg oseltamivir in children aged 1–2 years [65]. (**K**) Oral 45 mg oseltamivir in children aged 5 years [66]. Oral 60 mg oseltamivir in children aged (**L**) 7 years [66] and (**M**) 6 years [67]. (**N**) Oral 90 mg oseltamivir in children aged 3 years [67]. (**O**) Oral 0.14 mg/kg enalapril in children aged 2–24 months [68]. (**P**) Oral 0.13 mg/kg enalapril in children aged 3–5 years [68]. (**Q**) Oral 0.11 mg/kg enalapril in children aged 6–11 years [68]. (**R**) Oral 0.07 mg/kg enalapril in children aged 12–15 years [68]. Dots represent the observed concentrations. The solid lines indicate the mean predicted concentrations, and the shaded regions correspond to the 5th–95th percentile ranges.

**Figure 5:**
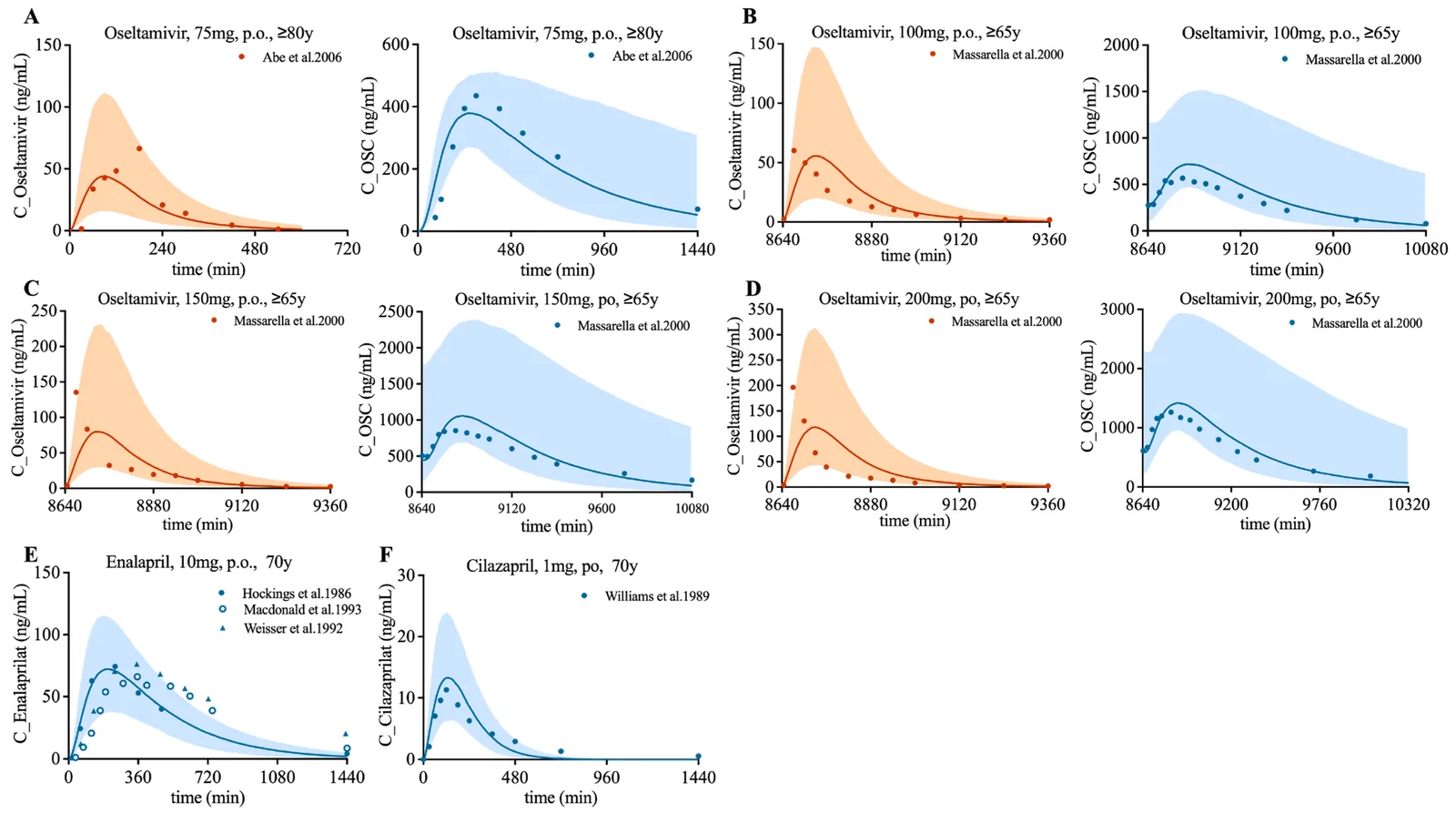
Plasma concentration–time profiles of oseltamivir, cilazapril, enalapril (orange), and their active metabolites (blue) in the geriatric population. (**A**) Oral 75 mg oseltamivir in the elderly aged ≥ 80 years [53]. (**B**) Oral 100 mg oseltamivir in the elderly aged ≥ 65 years [69]. (**C**) Oral 150 mg oseltamivir in the elderly aged ≥ 65 years [69]. (**D**) Oral 200 mg oseltamivir in the elderly aged ≥ 65 years [69]. (**E**) Oral 10 mg enalapril in the elderly aged 70 years [55,70,71]. (**F**) Oral 1 mg cilazapril in the elderly aged 70 years [43]. Dots represent the observed concentration. The solid lines indicate the mean predicted concentrations, and the shaded regions correspond to the 5th–95th percentile ranges.

**Figure 6:**
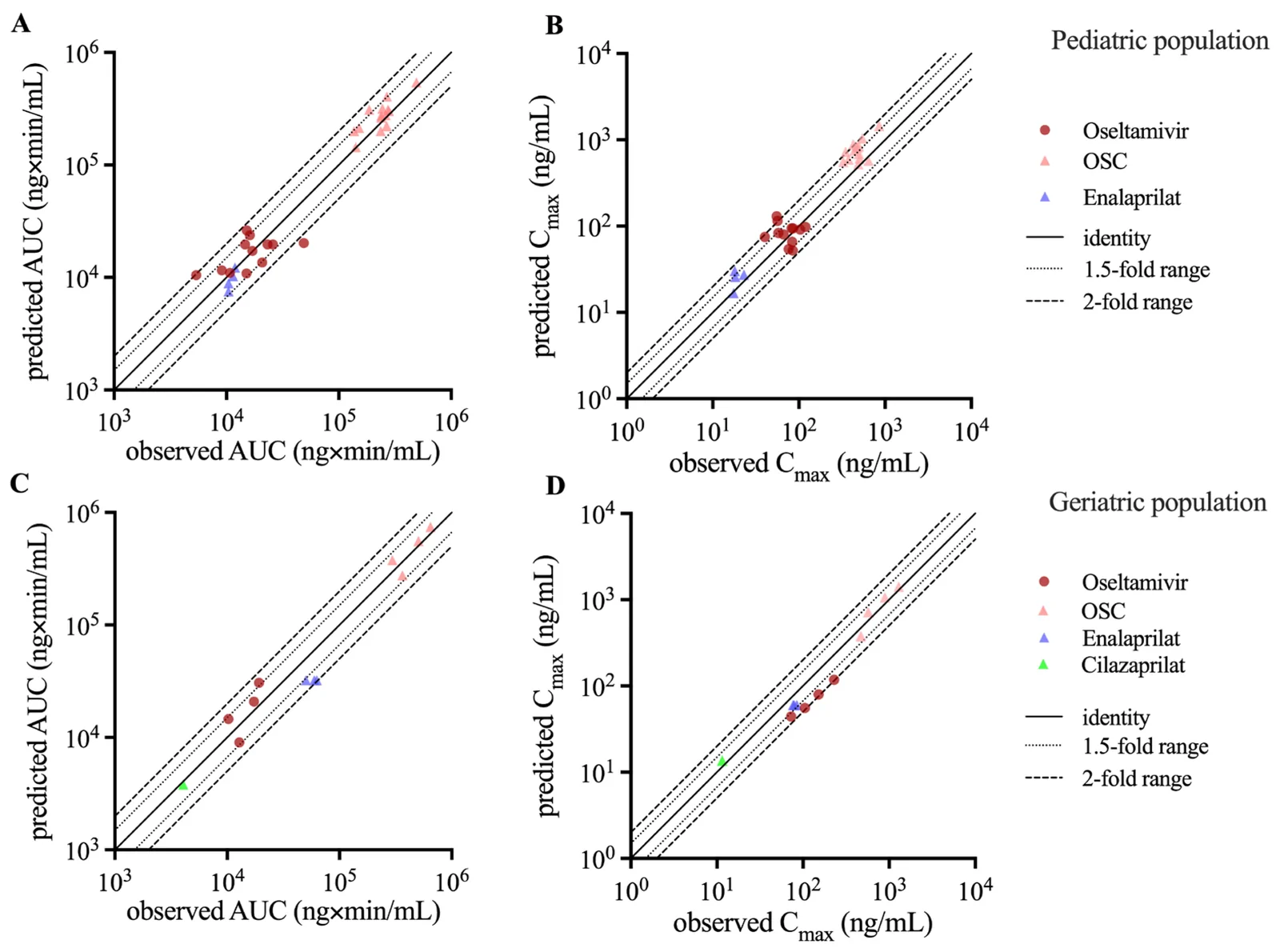
Goodness-of-fit plots showing predicted versus observed AUC and $C_{max}$ in pediatric (**A**,**B**) and geriatric (**C**,**D**) populations. The solid line represents the line of identity, and the dotted and dashed lines indicate the 1.5-fold and 2-fold error ranges. AUC: area under the plasma concentration-time curve; $C_{max}$: maximum plasma concentration.

**Figure 7:**
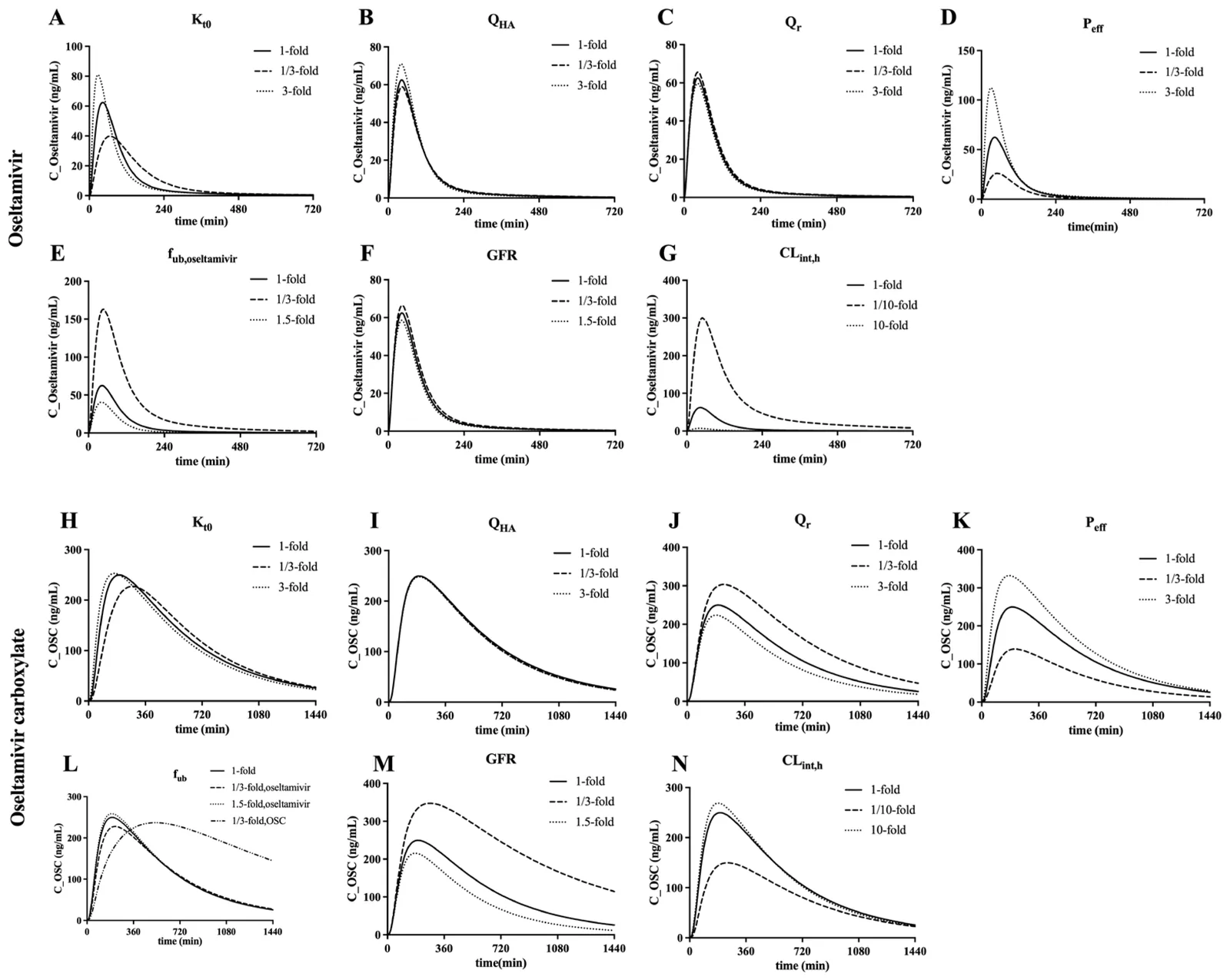
Sensitivity analysis following oral 75 mg oseltamivir in adults. Effect of changes in $K_{t0}$ (**A**), $Q_{HA}$ (**B**), $Q_{r}$ (**C**), $P_{eff}$ (**D**), $f_{ub}$ (**E**), GFR (**F**), and ${CL}_{int,h}$ (**G**) on plasma oseltamivir; effect of changes in $K_{t0}$ (**H**), $Q_{HA}$ (**I**), $Q_{r}$ (**J**), $P_{eff}$ (**K**), $f_{ub}$ (**L**), GFR (**M**), and ${CL}_{int,h}$ (**N**) on plasma oseltamivir carboxylate.

**Table 1:** Physiological parameters used in the adult PBPK model.

Tissue	Volume (mL) [22]	Blood Flow (mL/min) [22]
Lung	935	5897
Heart	318	251 [23]
Brain	1416	771
Muscle	25,948	708
Adipose	10,280	247 [23]
Skin	2777	330
Kidney	295	1202
Spleen	169	166
Stomach	146	81
Liver	1638	1664 ^a^
Hepatic artery	/	476
Venous	3552 ^b^	/
Artery	1776 ^b^	/
Rest of body	19,865 ^c^	740 ^d^
Duodenum	112	96
Jejunum	333	360
Ileum	222	204
Cecum	159	34
Colon	159	230

^a^: The blood flow rate of the liver was the sum of the blood flow rates of the hepatic artery, gastrointestinal tract, and spleen. ^b^: The volumes of the artery and vein were assumed to be 1:2. ^c^: The volume of the rest of the body equaled the total body volume (body weight = 70.1 kg; see Table S4) minus the summed volume of the listed organs. ^d^: The blood flow rate of the rest of the body equaled the cardiac output minus the summed blood flow rate of the other listed organs.

**Table 2:** Drug-specific input parameters for the adult PBPK model.

Parameters	Oseltamivir	OSC	Enalapril	Enalaprilat	Cilazapril	Cilazaprilat
Physiochemistry and protein binding						
MW (g/mol)	312.40	284.35	376.45	348.39	417.50	389.45
logP	1.3 ^a^	−1.3 ^a^	0.59 ^a^	−0.74 [34]	0.22 ^a^	−0.48 ^a^
pKa	7.7 [35]	4.19 ^a^	5.20 ^a^	2.03 [34]	3.4 ^a^	3.17 ^a^
$f_{up}$	0.58 [35]	0.97 [35]	0.50 [34]	0.68 [34]	0.70 [36]	0.76 [21]
$R_{b}$	1 ^b^	1 ^b^	0.74 [37]	0.73 [37]	1 ^b^	1 ^b^
Absorption						
$P_{eff}$ (10^−4^ cm/s)	2.43 [38] ^c^	/	1.6 [39] ^c^	/	1.27 ^d^	/
Elimination						
${CL}_{int,h}$ (mL/min)	11,776 ^e^	/	784 [16]	/	199.7 [16]	/
${CL}_{int,r}$ (mL/min)	638.2 [35] ^f^	760.1 [40] ^f^	382.3 [41] ^f^	284.6 [20] ^f^	118.3 [42] ^f^	175.4 [43] ^f^
Distribution ($K_{t:p}$) [33] ^g^						
Adipose	5.344	0.507	0.351	0.254	0.114	0.119
Brain	1.077	1.476	1.045	0.625	0.301	0.322
Muscle	0.903	1.879	1.689	0.573	0.277	0.296
Liver	0.928	1.154	0.844	0.536	0.298	0.316
Lung	0.960	2.575	2.399	0.812	0.423	0.441
Kidney	0.894	1.274	0.908	0.588	0.365	0.385
Spleen	0.903	1.597	1.199	0.679	0.335	0.356
Heart	0.887	1.393	1.032	0.585	0.398	0.419
Skin	1.048	1.59	1.11	0.716	0.432	0.445
Intestines	1.038	1.471	1.23	0.541	0.377	0.396
Stomach	0.903	1.471	1.23	0.541	0.377	0.396

OSC, oseltamivir carboxylate; MW, molecular weight; logP, the log of octanol/water partition coefficient; pKa, acid dissociation constant; $f_{up}$, unbound fraction in plasma; $R_{b}$, blood plasma concentration ratio; $P_{eff}$, human effective permeability; ${Cl}_{int,h}$, intrinsic hepatic clearance; ${Cl}_{int,r}$, intrinsic renal clearance; $K_{t:p}$, tissue to plasma partition coefficient. ^a^: Values were from the DrugBank database (www.drugbank.com). ^b^: Value was assumed to be 1. ^c^: Calculated from Papp using Equation (S5). ^d^: Fitted using pharmacokinetic data from a 2.5 mg oral dose of cilazapril [21]. ^e^: Value was calculated from the clearance in the S9 fraction (67 µL/min/mg protein) [10]. The total amount of S9 protein in the liver is 175,757 mg, calculated by multiplying S9 protein per gram of liver (107.3 mg/g [44]) by the total liver weight (1638 g). ^f^: Back-calculated from ${Cl}_{r}$ using Equation (S12). ^g^: Calculated using the Rodgers–Rowland method.

**Table 3:** Prediction performance of oseltamivir, enalapril, cilazapril, and their metabolites in adults.

Drug	Values	$C_{max}$	${AUC}_{0-t}$
Oseltamivir	AFE	1.15	1.00
	PE (%)	16.93	12.73
	GMFE	1.13	1.07
OSC	AFE	0.98	0.92
	PE (%)	13.32	8.61
	GMFE	1.07	1.08
Enalapril	AFE	0.85	1.41
	PE (%)	13.07	32.25
	GMFE	1.07	1.20
Enalaprilat	AFE	1.01	1.02
	PE (%)	12.69	17.07
	GMFE	1.07	1.13
Cilazapril	AFE	0.85	0.85
	PE (%)	14.34	14.65
	GMFE	1.02	1.03
Cilazaprilat	AFE	1.04	1.35
	PE (%)	13.68	28.51
	GMFE	1.11	1.14

Absolute fold error (AFE), prediction error (PE), and geometric mean fold error (GMFE).

**Table 4:** Prediction performance of oseltamivir, enalapril, cilazapril, and their metabolites in pediatric and geriatric populations.

Drug	Values	Pediatric Population		Geriatric Population	
		$C_{max}$	${AUC}_{0-t}$	$C_{max}$	${AUC}_{0-t}$
Oseltamivir	AFE	1.14	1.01	0.54	1.18
	PE (%)	31.03	23.32	45.53	34.93
	GMFE	1.23	1.21	1.84	1.40
OSC	AFE	1.57	1.17	1.07	1.05
	PE (%)	49.43	16.11	16.95	17.72
	GMFE	1.18	1.13	1.19	1.21
Enalaprilat	AFE	1.28	0.87	0.90	0.67
	PE (%)	20.78	10.71	10.39	31.53
	GMFE	1.18	1.09	1.11	1.48
Cilazaprilat	AFE	/	/	1.19	0.94
	PE (%)	/	/	18.80	6.16
	GMFE	/	/	1.19	1.07

Absolute fold error (AFE), prediction error (PE), and geometric mean fold error (GMFE).

## Data Availability

The original contributions presented in this study are included in the article/Supplementary Materials. Further inquiries can be directed to the corresponding author.

## Supplementary Materials

The following supporting information can be downloaded at: https://www.mdpi.com/article/10.3390/pharmaceutics18101211/s1, S1. CES1-mediated prodrug-metabolite PBPK model development [33,72,73,74]; Table S1. Relationship between organ mass (kg) and age in subjects aged 0–2 years [22]; Table S2. Relationship between organ mass (kg) and age in subjects aged 2–20 years [22]; Table S3. Relationship between organ blood flow rates and age in subjects aged 0–20 years [22,23]; Table S4. Relationship between body weight or height and age in subjects aged 0–20 years [22]; Table S5. Organ mass, organ blood flow rates, body weight, and height used in the geriatric PBPK model [24,25]; Table S6. Summary of clinical studies used in the PBPK model [19,20,21,43,51,52,53,54,55,56,57,58,59,60,61,62,63,64,65,66,67,68,69,70,71]; Figure S1: Predicted area under the plasma concentration-time curve (AUC) of oseltamivir carboxylate (OSC) in young adult and age-stratified elderly cohorts (aged 60, 70, and 80 years) following a single 75 mg oral dose of oseltamivir; Figure S2. Sensitivity analysis following a single 2 mg/kg oral dose of oseltamivir administered to 1-month-old infants. Effect of changes in $K_{t0}$ (A), $Q_{HA}$ (B), $Q_{r}$ (C), $P_{eff}$ (D), $f_{ub}$ (E), GFR (F) and ${CL}_{int,h}$ (G) on plasma oseltamivir; Effect of changes in $K_{t0}$ (H), $Q_{HA}$ (I), $Q_{r}$ (J), $P_{eff}$ (K), $f_{ub}$ (L), GFR (M) and ${CL}_{int,h}$ (N) on plasma oseltamivir carboxylate (OSC); Figure S3. Sensitivity analysis following a single 75-mg oral dose of oseltamivir administered to 80-year-old geriatric subjects. Effect of changes in $K_{t0}$ (A), $Q_{HA}$ (B), $Q_{r}$ (C), $P_{eff}$ (D), $f_{ub}$ (E), GFR (F) and ${CL}_{int,h}$ (G) on plasma oseltamivir; Effect of changes in $K_{t0}$ (H), $Q_{HA}$ (I), $Q_{r}$ (J), $P_{eff}$ (K), $f_{ub}$ (L), GFR (M) and ${CL}_{int,h}$ (N) on plasma oseltamivir carboxylate (OSC); Figure S4. Sensitivity analysis following a single 10 mg oral dose of enalapril administered to adults. Effect of changes in $K_{t0}$ (A), $Q_{HA}$ (B), $Q_{r}$ (C), $P_{eff}$ (D), $f_{ub}$ (E), GFR (F) and ${CL}_{int,h}$ (G) on plasma enalapril; Effect of changes in $K_{t0}$ (H), $Q_{HA}$ (I), $Q_{r}$ (J), $P_{eff}$ (K), $f_{ub}$ (L), GFR (M) and ${CL}_{int,h}$ (N) on plasma enalaprilat; Figure S5. Sensitivity analysis following a single 2.5 mg oral dose of cilazapril administered to adults. Effect of changes in $K_{t0}$ (A), $Q_{HA}$ (B), $Q_{r}$ (C), $P_{eff}$ (D), $f_{ub}$ (E), GFR (F) and ${CL}_{int,h}$ (G) on plasma cilazapril; Effect of changes in $K_{t0}$ (H), $Q_{HA}$ (I), $Q_{r}$ (J), $P_{eff}$ (K), $f_{ub}$ (L), GFR (M) and ${CL}_{int,h}$ (N) on plasma cilazaprilat. Figure S6. Impact of different coefficient of variation (CV) assumptions on simulated concentration-time profiles of oseltamivir and oseltamivir carboxylate (OSC). Solid lines represent the mean predicted concentration-time profiles, and dotted lines indicate the corresponding 5th–95th percentile prediction intervals. Different colors represent different CV assumptions (green: 10% CV; black: 20% CV; red: 30% CV).

## Abbreviations

PBPK, physiologically based pharmacokinetic; CES1, carboxylesterase 1; AUC, area under the curve; $C_{max}$, maximum plasma concentration; ${CL}_{int,h}$, intrinsic hepatic clearance; ${CL}_{int,r}$, intrinsic renal clearance; $f_{ub}$, free fraction of drug in blood; $f_{up}$, free fraction of drug in plasma; $P_{eff}$, the effective permeability coefficient; $K_{t0}$, gastric emptying rate; $R_{b}$, blood-to-plasma concentration ratio; GFR, glomerular filtration rate; ALB, albumin; $Q_{HA}$, hepatic artery blood flow; $Q_{r}$, renal blood flow; BW, body weight; BSA, body surface area; OSC, oseltamivir carboxylate.
