# Supplementary material for: PBPK Modeling of CES1-Activated Prodrugs Across Different Age Groups: Simultaneous Prodrug–Metabolite Prediction for Oseltamivir, Enalapril, and Cilazapril
Source: Pharmaceutics. 2026 Sep 23;18(10):1211. doi: 10.3390/pharmaceutics18101211 (PMC13708068; doi:10.3390/pharmaceutics18101211)
Supplement: Supplementary file 1 [file pharmaceutics-18-01211-s001.zip › pharmaceutics-4489477-supplementary.pdf]

Supplementary Information

PBPK Modeling of CES1-Activated Prodrugs Across Different Age Groups: Simultaneous Prodrug–Metabolite Prediction for Oseltamivir, Enalapril, and Cilazapril

S1. CES1-mediated prodrug-metabolite PBPK model development

A whole-body PBPK model was constructed to describe the pharmacokinetics of oseltamivir, cilazapril and enalapril and their active metabolites in healthy subjects. The developed PBPK model comprises 13 compartments, including arterial blood, venous blood, lungs, heart, brain, muscle, adipose tissue, skin, liver, kidneys, spleen, gut, and rest of the body. The blood perfusion-rate limited model was used to illustrate organ compartments. The liver and kidneys were designated as the primary organs responsible for drug elimination.

In general tissue compartments (t):

$$V_t \frac{dC_t}{dt} = Q_t \times \left(C_{art} - \frac{C_t}{K_{t:p}/R_b}\right) \tag{S1}$$

where V_t, C_t, Q_t and $K_{t:p}$ represent the volume of tissue or organ, drug concentration, blood flow rate, and ratio of drug concentration in tissue to plasma, respectively. C_{art} is drug concentrations in arterial blood. R_b represents ratio of drug concentration in blood to plasma. $K_{t:p}$ values were calculated using the Rodgers–Rowland method [1].

It is assumed that absorption occurs in the duodenum, jejunum, and ileum. The gastrointestinal absorption of the ester prodrugs was mathematically characterized using segmented intestinal absorption rate constants. Notably, while gastric acidity varies across different age groups, the intraluminal pH profiles of the small intestinal segments (duodenum, jejunum, and ileum) are known to remain constant and comparable among pediatric populations, adults, and geriatric populations due to the robust buffering capacity of pancreatic bicarbonate secretion [2,3]. Thus, standard adult-derived intestinal pH values were consistently maintained across the lifespan cohorts to avoid model overparameterization. The disposition of prodrugs in the stomach, intestinal lumen, and intestinal wall is illustrated as follows.

In the stomach (A_0):

$$\frac{dA_0}{dt} = -K_{t,0} \times A_0 \tag{S2}$$

where $K_{t,0}$ is the constant of stomach emptying rate, A_0 represents the amount of drug in the stomach.

In the gut lumen (A_i):

$$\frac{dA_i}{dt} = K_{t,i-1} \times A_{i-1} - K_{t,i} \times A_i - k_{a,i} \times A_i \tag{S3}$$

where i=1, 2, 3 represent duodenum, jejunum and ileum, respectively. A_i, $K_{t,i}$, and $k_{a,i}$ is the drug amount in the gut lumen, the transit rate constant of the i-th intestinal lumen, and the rate constant of drug absorption in the i-th intestinal lumen. $k_{a,i}$ can be estimated using the equations:

$$k_{a,i} = \frac{2 \times P_{eff}}{r_i} \tag{S4}$$

$$logP_{eff} = 0.4926 \times logP_{app,Caco-2} - 0.1454 \tag{S5}$$

where P_{eff} and r_i denotes human effective permeability and radius of the i-th gut lumen. $P_{app,Caco-2}$ is apparent permeability coefficient in the apical to basolateral direction across Caco-2 monolayer [4]:

In the gut wall (A_{gwi}):

$$\frac{dA_{gwi}}{dt} = Q_{gwi} \times (C_{art} - \frac{\frac{A_{gwi}}{V_{gwi}}}{\frac{K_{gut:p}}{R_b}}) + k_{a,i} \times A_i \tag{S6}$$

where A_{gwi}, Q_{gwi}, V_{gwi} and $K_{gut:p}$ denote drug amount in gut wall, blood flow rate, volume of the i-th intestinal region, and ratio of drug concentration in gut wall to plasma. For the cecum and colon, where absorption is not considered, the term $k_{a,i} \times A_i$ is excluded.

In the liver (h):

It was assumed that activation of the prodrug occurs exclusively in the liver.

For prodrug (pro):

$$V_h \times \frac{dC_{h,pro}}{dt} = Q_{HA} \times C_{art} + Q_{spl} \times \frac{C_{spl,pro}}{K_{spl:p,pro}/R_{b,pro}} + \sum_{i=0}^{5}(Q_{gwi} \times \frac{C_{gwi,pro}}{K_{gut:p,pro}/R_{b,pro}}) - \left(Q_{HA} + Q_{spl} + \sum_{i=0}^{5} Q_{gwi}\right) \times \frac{C_{h,pro}}{K_{h:p,pro}/R_{b,pro}} - PBSF \times CL_{int,CES1} \times \frac{C_{h,pro} \times f_{ub,pro}}{K_{h:p,pro}/R_{b,pro}} \tag{S7}$$

For active metabolite (met):

$$V_{hep} \times \frac{dC_{hep,met}}{dt} = Q_{HA} \times C_{art,met} + Q_{spl} \times \frac{C_{spl,met}}{K_{spl:p,met}/R_{b,met}} + \sum_{i=0}^{5}(Q_{gwi} \times \frac{C_{gwi,met}}{K_{gut:p,met}/R_{b,met}}) + \frac{MW_{met}}{MW_{pro}} \times PBSF \times CL_{int,CES1} \times \frac{C_{h,pro} \times f_{ub,pro}}{K_{h:p,pro}/R_{b,pro}} - \left(Q_{HA} + Q_{spl} + \sum_{i=0}^{5} Q_{gwi}\right) \times \frac{C_{h,met}}{K_{h:p,met}/R_{b,met}} \tag{S8}$$

where Q_{HA} and Q_{spl} are blood flow rates of hepatic artery (HA) and spleen (spl), respectively. V_h represents the volume of liver. MW is molecular weight. The unbound fraction in blood (f_{ub}) was obtained by calculating f_{up}/R_b. PBSF represents the total amount of liver microsomal protein. $CL_{int,CES1}$ is the intrinsic hepatic clearance of prodrugs, which can be estimated using in vitro hepatic microsomes experiment or back-calculated from total hepatic clearance (CL_h) using equations:

$$CL_{int,CES1} = \frac{V_{max,CES1}}{K_{m,CES1}} \tag{S9}$$

where $V_{max,CES1}$ and $K_{m,CES1}$ represent the maximum velocity and Michaelis-Menten constant of CES1-mediated metabolism, respectively.

or

$$CL_h = \frac{Q_h \times f_{ub} \times CL_{int,CES1}}{Q_h + f_{ub} \times CL_{int,CES1}} \quad \text{(S10)}$$

In the kidneys (r):

Oseltamivir, cilazapril, and enalapril are partially eliminated through the kidneys, with their active metabolites primarily eliminated via the kidneys.

$$\frac{V_r \times dC_r}{dt} = Q_r \times C_{art} - (Q_r + f_{ub} \times CL_{int,r}) \times \frac{C_r}{K_{r:p}/R_b} \quad \text{(S11)}$$

where Q_r and V_r represent the renal blood flow and renal volume, respectively. $CL_{int,r}$ is the renal intrinsic clearance. $CL_{int,r}$ can be back-calculated by the total clearance of the kidneys (CL_r):

$$CL_r = \frac{Q_r \times f_{ub} \times CL_{int,r}}{Q_r + f_{ub} \times CL_{int,r}} \quad \text{(S12)}$$

In the artery (art) and vein (ven)：

$$V_{art} \frac{dC_{art}}{dt} = Q_{total} \times \left(\frac{C_{lun}}{K_{lun:p}/R_b} - C_{art} \right) \quad \text{(S13)}$$

$$V_{ven} \frac{dC_{ven}}{dt} = \Sigma_t \left(Q_t \times \frac{C_t}{K_{t:p}/R_b} \right) - Q_{total} \times C_{ven} \quad \text{(S14)}$$

where Q_{total} represents the cardiac output. Lun indicates lung.

Table S1. Relationship between organ mass (kg) and age in subjects aged 0–2 years [5].

Tissue	Organ mass-age equation (0-2years)
Lung	$(0.0524 + 0.548 \times age)/(1 + 2.56 \times age - 0.320 \times age^2)$
Heart	$(0.0207 + 0.0473 \times age)/(1 + 0.465 \times age - 0.0161 \times age^2)$
Brain	$(0.347 + 0.630 \times age)/(1 - 0.106 \times age + 0.171 \times age^2)$
Muscle	$(0.78 + 1.7 \times age)/(1 + 0.553 \times age - 0.0908 \times age^2)$
Adipose	$(0.327 + 8.63 \times age)/(1 + 2.21 \times age + 0.323 \times age^2)$
Skin	$(0.171 + 0.563 \times age)/(1 + 1.25 \times age - 0.0624 \times age^2)$
Kidney	$(0.0302 + 0.0459 \times age)/(1 - 0.0449 \times age + 0.0997 \times age^2)$
Spleen	$(0.0098 + 0.0831 \times age)/(1 + 2.56 \times age - 0.310 \times age^2)$
Stomach	$(0.00675 + 0.0531 \times age)/(1 + 2.11 \times age - 0.335 \times age^2)$
Liver	$(0.125 + 0.481 \times age)/(1 + 0.956 \times age - 0.0694 \times age^2)$
Intestine	$(0.0447 + 0.109 \times age)/(1 + 0.107 \times age + 0.0556 \times age^2)$
Blood volume	$(0.231 + 0.669 \times age)/(1 + 0.29 \times age + 0.0177 \times age^2)$

Table S2. Relationship between organ mass (kg) and age in subjects aged 2–20 years [5].

Tissue	Organ mass-age equation (2-20years)
Lung	$(0.216 - 0.00404 \times age)/(1 - 0.0769 \times age + 0.00169 \times age^2)$
Heart	$(0.0507 + 0.000507 \times age)/(1 - 0.0814 \times age + 0.00204 \times age^2)$
Brain	$(0.583 + 0.967 \times age)/(1 + 0.656 \times age - 0.000210 \times age^2)$
Muscle	$(1.01 + 0.566 \times age)/(1 - 0.0662 \times age + 0.00197 \times age^2)$
Adipose	$(1.89 + 0.0387 \times age)/(1 - 0.0789 \times age + 0.00209 \times age^2)$
Skin	$(0.297 + 0.00120 \times age)/(1 - 0.0916 \times age + 0.00237 \times age^2)$
Kidney	$(0.0788 - 0.000526 \times age)/(1 - 0.07 \times age + 0.00156 \times age^2)$
Spleen	$(0.0281 + 0.00222 \times age)/(1 - 0.0503 \times age + 0.00103 \times age^2)$
Stomach	$(0.0165 + 0.005 \times age)/(1 - 0.0389 \times age + 0.00139 \times age^2)$
Liver	$(0.315 + 0.0350 \times age)/(1 - 0.0272 \times age + 0.000319 \times age^2)$
Intestinal tract	$(0.0840 + 0.0463 \times age)/(1 - 0.0118 \times age + 0.000563 \times age^2)$
Blood Volume	$(0.760 + 0.0168 \times age)/(1 - 0.0775 \times age + 0.00187 \times age^2)$

Table S3. Relationship between organ blood flow rates and age in subjects aged 0–20 years [5].

Tissue (mL/min/100g organ)	Organ blood flow and age equations（0-20year）
Muscle	$(4.73 - 0.189 \times age)/(1 + 0.0672 \times age - 0.00515 \times age^2)$
Skin	$(9.65 + 25.7 \times age)/(1 + 1.04 \times age + 0.0585 \times age^2)$
Kidney	$(229 + 700 \times age)/(1 + 1.49 \times age + 0.00108 \times age^2)$
Spleen	$(223 + 102 \times age)/(1 + 1.03 \times age + 0.00400 \times age^2)$
Stomach	$(128 + 63.9 \times age)/(1 + 1.11 \times age + 0.00568 \times age^2)$
Liver	$(25.6 + 2.33 \times age)/(1 - 0.0227 \times age + 0.00527 \times age^2)$
Intestinal tract	$(216 + 106 \times age)/(1 + 1.09 \times age + 0.00562 \times age^2)$
Lung (L/min)	$(0.589 + 1.23 \times age)/(1 + 0.283 \times age - 0.00641 \times age^2)$
Brain	$(54.0 + 73.6 \times age)/(1 + 0.382 \times age + 0.051 \times age^2)$
Heart [6]	$0.79 \times L/min/ kg\ organ$
Adipose [6]	$0.024 \times L/min/ kg\ organ$

Table S4. Relationship between body weight or height and age in subjects aged 0–20 years [5].

Age	Body weight/height-age equation
Body weight (kg)	
0-2 years	$(3.41 + 20.1 \times age)/(1 + 1.46 \times age - 0.107 \times age^2)$
2-20 years	$(9.86 + 0.370 \times age)/(1 - 0.0789 \times age + 0.00205 \times age^2)$
Height (cm)	
0-2 years	$(50.5 + 117 \times age)/(1 + 1.28 \times age - 0.0696 \times age^2)$

2-20 years	$(74.8 + 3.35 \times age)/(1 - 0.0354 \times age + 0.00125 \times age^2)$

Table S5. Organ mass, organ blood flow rates, body weight, and height used in the geriatric PBPK model [7].

Parameter	60 years		70 years		80 years	
	Weight (g)	Blood flow rate (mL/min)	Weight (g)	Blood flow rate (mL/min)	Weight (g)	Blood flow rate (mL/min)
Lung	1349.0	5329	1271.8	4731	1138.0	4213
Heart	454.6	299.1	464.8	308.7	441.4	334.7
Brain	1463.3	692.9	1434.3	650.6	1400.1	623.8
Muscle	29600.0	975.0	28562.1	922.3	26524.8	861.7
Adipose	17920.7	381.4	19447.4	408.0	19947.7	419.8
Skin	3692.0	319.2	3635.2	314.3	3537.0	305.8
Kidney	455.6	913.3	441.7	730.3	395.7	567.3
Spleen	197	142[a]	186.6	121[a]	160.4	108[a]
Stomach	150[b]	84[a]	148.1[b]	71[a]	141.8[b]	64[a]
Liver	2041.3	361.9	1714.6	278.5	1417.3	209.1
Vein(mL)	3559[c]	/	3471[c]	/	3385[c]	/
Artery(mL)	1779[c]	/	1735[c]	/	1693[c]	/
ROB	8326.9	370.9	7588.2	255.7	6960.8	119.3
Duodenum	115[b]	82[a]	113.6[b]	70[a]	108.8[b]	62[a]
Jejunum	342[b]	308[a]	337.8[b]	261[a]	323.5[b]	234[a]
Ileum	228[b]	174[a]	225.2[b]	148[a]	215.7[b]	132[a]
Caecum	163.3[b]	29[a]	161.3[b]	25[a]	154.5[b]	22[a]
Colon	163.3[b]	197[a]	161.3[b]	167[a]	154.5[b]	149[a]
Body weight (kg)	72		71.1		68.1	
Height (cm)	171.2		167.4		165.5	

a Blood flow rates were scaled from the total splanchnic blood flow values reported by Schlender et al [7].
b Organ masses were estimated from the adult data based on the body weight ratio.
c Blood volume was calculated using the formula $e^{(0.067 \times BSA - 0.0025 \times age + 1.7)}$ [8], with a venous to arterial volume ratio of 2:1.

Table S6. Summary of clinical studies used in the PBPK model

No	Reference	Dose	N,Gender	Age, years (mean ± SD, [median] (range))	Weight, kg (mean ± SD, [median] (range))	PK profiles	Group	Subjects' information	Role of dataset	Ref
Oseltamivir										
1	Jittamala et al.2014	75mg, po	4 M/8 F	[31.5] (22.0–45.0) y	[59.1] (51.0–71.7)	Oseltamivir, OSC	Adults (non-obese)	Healthy non-obese volunteers	Model development	[9]
		75mg, po	2 M/10 F	[37.0] (27.0–46.0) y	[87.3] (69.6–120.5)	Oseltamivir, OSC	Adults (obese)	Healthy obese volunteer	Internal validation	
		150mg, po	4 M/8 F	[31.5] (22.0–45.0) y	[59.1] (51.0–71.7)	Oseltamivir, OSC	Adults (non-obese)	Healthy non-obese volunteers	Internal validation	
		150mg, po	2 M/10 F	[37.0] (27.0–46.0) y	[87.3] (69.6–120.5)	Oseltamivir, OSC	Adults (obese)	Healthy obese volunteers	Internal validation	
2	Abe et al.2006	75mg, po	7 M	23±2.4 y	59.5±5.1	Oseltamivir, OSC	Adults	Healthy volunteers	External validation	[10]
		75mg, po	2 M/3 F	84±3.2 y	53.8±8.0	Oseltamivir, OSC	Geriatrics	Healthy volunteers	External validation	
3	Tarkiainen et al.2012	75mg, po	9 M/3 F	23±3 y	77±11	Oseltamivir, OSC	Adults	Healthy volunteers	External validation	[11]
4	Brennan et al.2012	75mg, po	23 M/1 F	31.3±8.49[33.0](18–52) y	74.70±10.08[72.20](55.8–90.0)	Oseltamivir, OSC	Adults	Healthy volunteers	External validation	[12]
		100 mg, iv. over 2h	23 M/1 F	31.3±8.49[33.0](18–52) y	74.70±10.08[72.20](55.8–90.0)	Oseltamivir, OSC	Adults	Healthy volunteers	External validation	
		200 mg, iv. over 2h	23 M/1 F	31.3±8.49[33.0](18–52) y	74.70±10.08[72.20](55.8–90.0)	Oseltamivir, OSC	Adults	Healthy volunteers	External validation	

		400 mg, iv. over 2h	23 M/1 F	31.3±8.49[33.0](18–52) y	74.70±10.08[72.20](55.8–90.0)	Oseltamivir, OSC	Adults	Healthy volunteers	External validation	
5	Snell et al.2005	75mg, po	11 NR	NR	NR	Oseltamivir, OSC	Adults	Healthy volunteers	External validation	[13]
6	Rath et al.2015	2mg/kg q12h for 5 days, po	3 M/2 F	25.2±5.36 [28.0] (18–30) d	3.72±0.47	Oseltamivir, OSC	Pediatrics	Influenza	External validation	[14]
		2.5mg/kg q12h for 5 days, po	12 M/8 F	57.0±17.24 [55.5] (32–84) d	4.89±0.93	Oseltamivir, OSC	Pediatrics	Influenza	External validation	
		3mg/kg q12h for 5 days, po	21 M/19F	223.2±79.41 [215.5] (92-349) d	7.64±1.77	Oseltamivir, OSC	Pediatrics	Influenza	External validation	
7	Kimberlin et al.2013	3mg/kg q12h for 5 days, po	15 M/8 F	1(0.43–2) m	[4.30] (3.27–5.60)	Oseltamivir, OSC	Pediatrics	Influenza	External validation	[15]
		3mg/kg q12h for 5 days, po	8 M/2 F	4(3–5) m	[6.20] (3.50–8.86)	Oseltamivir, OSC	Pediatrics	Influenza	External validation	
		3mg/kg q12h for 5 days, po	11 M/13 F	6(6–8) m	[7.35] (4.62–11.30)	Oseltamivir, OSC	Pediatrics	Influenza	External validation	
		3mg/kg q12h for 5 days, po	4 M/3 F	10(9–10) m	[8.50](7.30–10.70)	Oseltamivir, OSC	Pediatrics	Influenza	External validation	
		3.5mg/kg q12h, po	2 M/6 F	10(9–11) m	[8.50](5.10–10.40)	Oseltamivir, OSC	Pediatrics	Influenza	External validation	
		3.5mg/kg q12h for 5 days, po	2 M/1 F	16(13–21) m	[12.70](12.20–13.50)	Oseltamivir, OSC	Pediatrics	Influenza	External validation	
		30mg q12h for 5 days, po	9 M/3 F	16.5(12–22) m	[11.39](8.12–20.60)	Oseltamivir, OSC	Pediatrics	Influenza	External validation	
8	Tang et al.2019	45mg q12h, po	1 NR	5 y	15.2	Oseltamivir, OSC	Pediatrics	Status asthmaticus	External validation	[16]
		60mg, po	1 NR	7 y	25	Oseltamivir, OSC	Pediatrics	Respiratory failure	External validation	

9	Bautista et al.2019	60mg q12h, po	10 M/5 F	6(1–12) y	26.7(11.9)	OSC	Pediatrics	Influenza	External validation	[17]
		135mg q12h, po	11 M/4 F	3(2–8) y	18.9(7.8)	OSC	Pediatrics	Influenza	External validation	
10	Massarella et al.2000	100 mg for 7 days, q12h, po	18 NR	≥65 y	NR	Oseltamivir, OSC	Geriatrics	Healthy volunteers	External validation	[18]
		150 mg for 7 days, q12h, po	18 NR	≥65 y	NR	Oseltamivir, OSC	Geriatrics	Healthy volunteers	External validation	
		200 mg for 7 days, q12h, po	18 NR	≥65 y	NR	Oseltamivir, OSC	Geriatrics	Healthy volunteers	External validation	
Enalapril										
11	Ohnishi et al.1989	10mg, po	7 M	21-39 y	NR	Enalapril, Enalaprilat	Adults	Healthy volunteers	Model development	[19]
12	Marzo et al.2002	10mg, po	5 M/5 F	29±8.8(20–48) y	66±9.2(52–81)	Enalapril, Enalaprilat	Adults	Healthy volunteers	External validation	[20]
		20mg, po	5 M/5 F	29±8.8(20–48) y	66±9.2(52–81)	Enalapril, Enalaprilat	Adults	Healthy volunteers	External validation	
13	Baba et al. 1990	10mg, po	10 M	48.8±13.5 y	NR	Enalapril, Enalaprilat	Adults	Healthy volunteers	External validation	[21]
14	Moffett et al.2014	10 mg, po	45 NR	(18–55) y	≥50	Enalapril, Enalaprilat	Adults	Healthy volunteers	External validation	[22]
15	Portoles et al.2004	20 mg, po	12 M/12 F	22.8±2.2 (19–30) y	65.1±12.4	Enalapril, Enalaprilat	Adults	Healthy volunteers	External validation	[23]
16	Hersh et al.1996	20 mg, po	6M	50.8 (44–55) y	75.3	Enalaprilat	Adults	Hypertensive patients with normal renal function	External validation	[24]
17	Macdonald et al.1993	10 mg, po	9 M	(21–39) y	NR	Enalaprilat	Adults	Healthy volunteers	External validation	[25]

		10 mg, po	6 M/3 F	(66–96) y	NR	Enalaprilat	Geriatrics	Healthy volunteers	External validation	
18	Hockings et al.1986	10mg, po	6 M/3 F	69±4 (65–73) y	70±13	Enalaprilat	Geriatrics	Healthy volunteers	External validation	[26]
19	Weisser et al. 1992	10 mg, po	7 F/12 M	(62–84) y	NR	Enalaprilat	Geriatrics	Hypertensive patients	External validation	[27]
20	Wells et al.2001	0.14 mg/kg, po	6 M/3 F	(2m–2y)	(3.5–13.8)	Enalaprilat	Pediatrics	Hypertensive patients	External validation	[28]
		0.13 mg/kg, po	5 M/4 F	(3–5)y	(11.4–25.9)	Enalaprilat	Pediatrics	Hypertensive patients	External validation	
		0.11 mg/kg, po	6 M/4 F	(6–11) y	(23.1–59.5)	Enalaprilat	Pediatrics	Hypertensive patients	External validation	
		0.07 mg/kg, po	7 M/5 F	(12–15) y	(38.7–160.6)	Enalaprilat	Pediatrics	Hypertensive patients	External validation	
Cilazapril										
21	Williams et al.1989(1)	2.5mg, po	12 M	24.1(19–38) y	74 (65–82)	Cilazapril, Cilazaprilat	Adults	Healthy volunteers	Model development	[29]
22	Massarella et al.1989	0.5mg, po	24 NR	29(20–47) y	74(50–93)	Cilazapril, Cilazaprilat	Adults	Healthy volunteers	External validation	[30]
		1mg, po	24 NR	29(20–47) y	74(50–93)	Cilazapril, Cilazaprilat	Adults	Healthy volunteers	External validation	
		2.5mg, po	24 NR	29(20–47) y	74(50–93)	Cilazapril, Cilazaprilat	Adults	Healthy volunteers	External validation	
		5mg, po	24 NR	29(20–47) y	74(50–93)	Cilazapril, Cilazaprilat	Adults	Healthy volunteers	External validation	
23	Nilsen et al.1989	5mg, po	9 M	(24–35) y	NR	Cilazaprilat	Adults	Healthy volunteers	External validation	[31]

24	Williams et al.1989(2)	1mg, po	9 M/3 F	22.2(18–31) y	64.8 (50.0–81.0)	Cilazaprilat	Adults	Healthy volunteers	External validation	[32]
		1mg, po	9 M/3 F	69.1(65–83) y	73.9(58.0–96.8)	Cilazaprilat	Geriatrics	Healthy volunteers	External validation	
25	Anderson et al.1996	2.5mg, po	7 M/6 F	31(23–44) y	67.8(50–99)	Cilazaprilat	Adults	Healthy volunteers	External validation	[33]

M: male; F: female; d, day; m, month; y, year; NR: not reported.

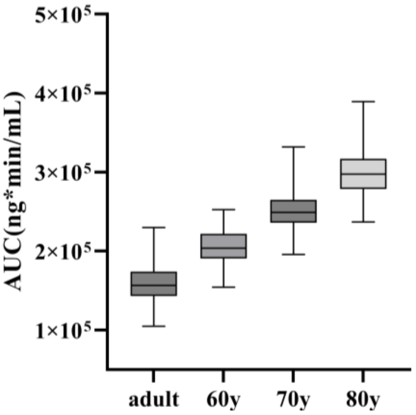

Figure S1: Predicted area under the plasma concentration-time curve (AUC) of oseltamivir carboxylate (OSC) in young adult and age-stratified elderly cohorts (aged 60, 70, and 80 years) following a single 75mg oral dose of oseltamivir.

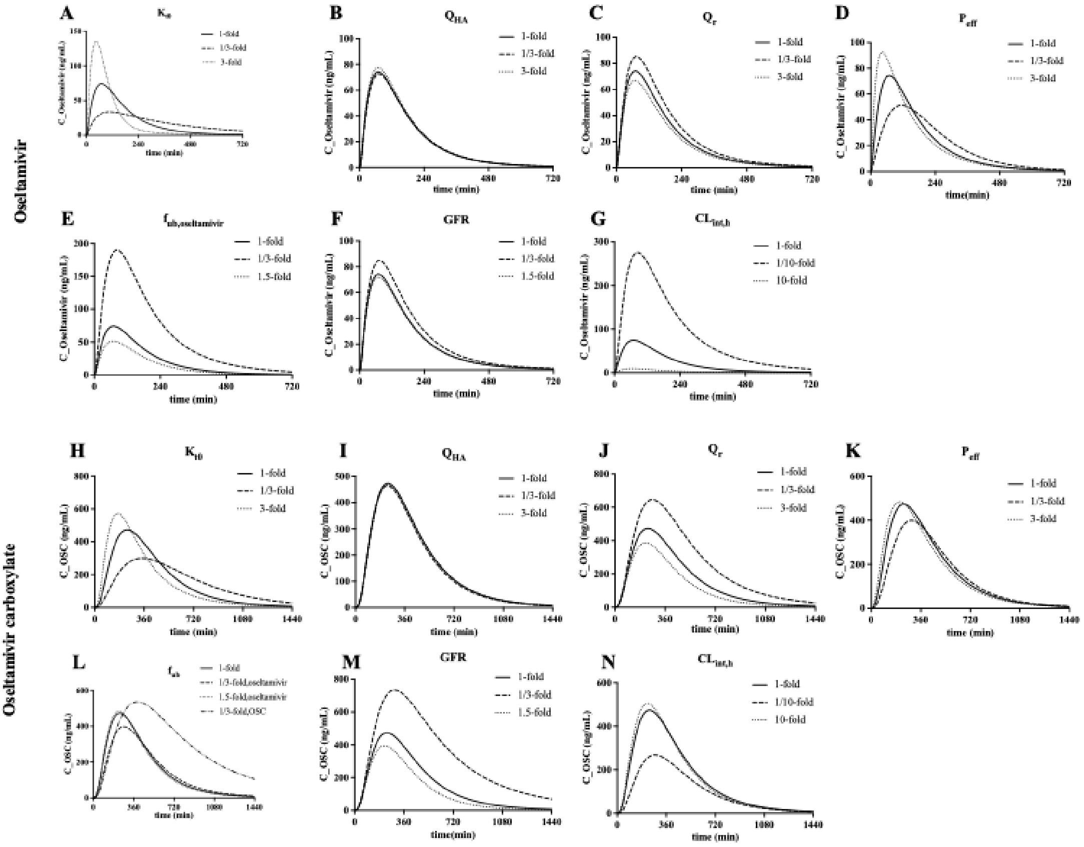

Figure S2. Sensitivity analysis following a single 2 mg/kg oral dose of oseltamivir administered to 1-month-old infants. Effect of changes in K_{t0} (A), Q_{HA} (B), Q_r (C), P_{eff} (D), f_{ub} (E), GFR (F) and $CL_{int,h}$ (G) on plasma oseltamivir; Effect of changes in K_{t0} (H), Q_{HA} (I), Q_r (J), P_{eff} (K), f_{ub} (L), GFR (M) and $CL_{int,h}$ (N) on plasma oseltamivir carboxylate (OSC).

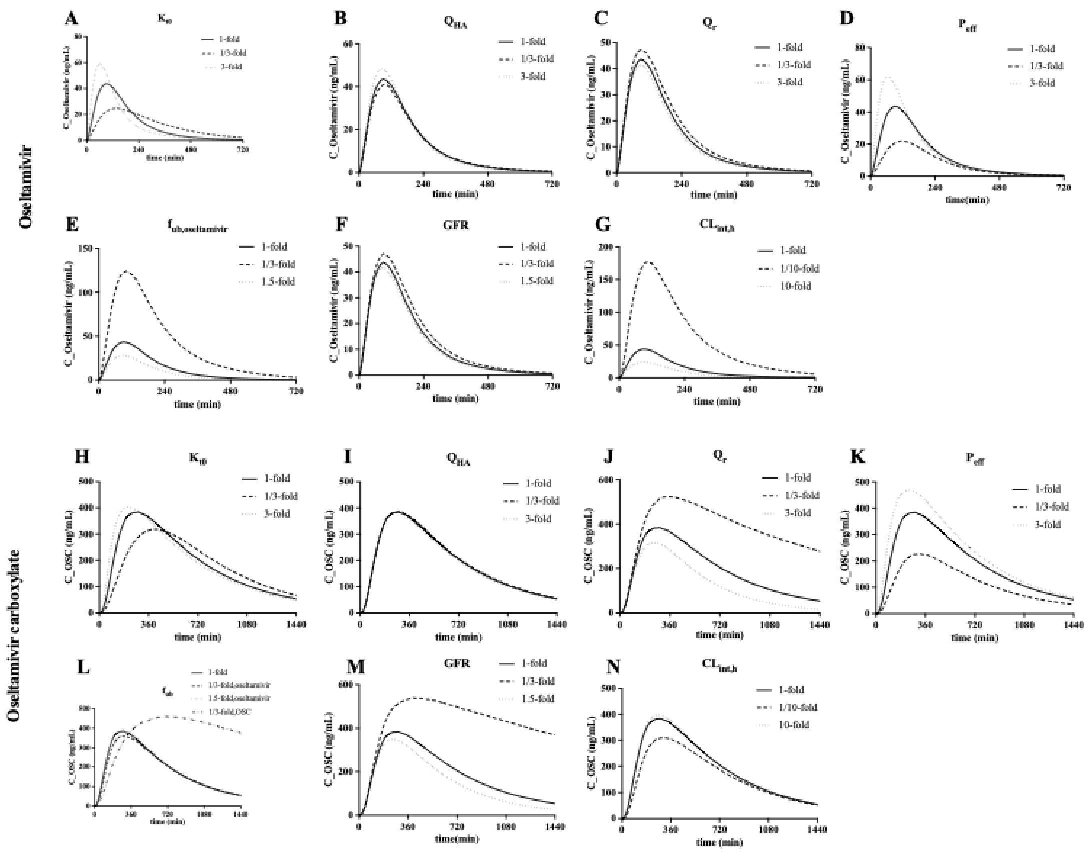

Figure S3. Sensitivity analysis following a single 75-mg oral dose of oseltamivir administered to 80-year-old geriatric subjects. Effect of changes in K_{t0} (A), Q_{HA} (B), Q_r (C), P_{eff} (D), f_{ub} (E), GFR (F) and $CL_{int,h}$ (G) on plasma oseltamivir; Effect of changes in K_{t0} (H), Q_{HA} (I), Q_r (J), P_{eff} (K), f_{ub} (L), GFR (M) and $CL_{int,h}$ (N) on plasma oseltamivir carboxylate (OSC).

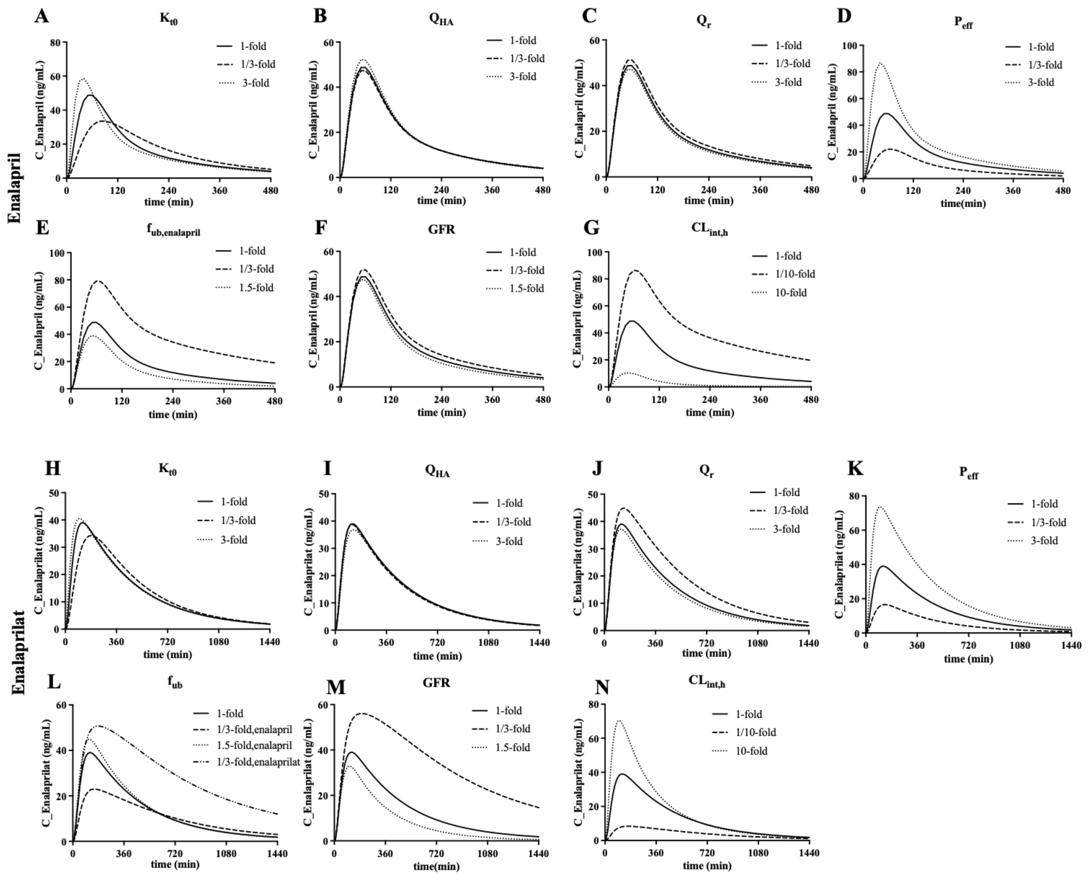

Figure S4. Sensitivity analysis following a single 10mg oral dose of enalapril administered to adults. Effect of changes in K_{t0} (A), Q_{HA} (B), Q_r (C), P_{eff} (D), f_{ub} (E), GFR (F) and $CL_{int,h}$ (G) on plasma enalapril; Effect of changes in K_{t0} (H), Q_{HA} (I), Q_r (J), P_{eff} (K), f_{ub} (L), GFR (M) and $CL_{int,h}$ (N) on plasma enalaprilat.

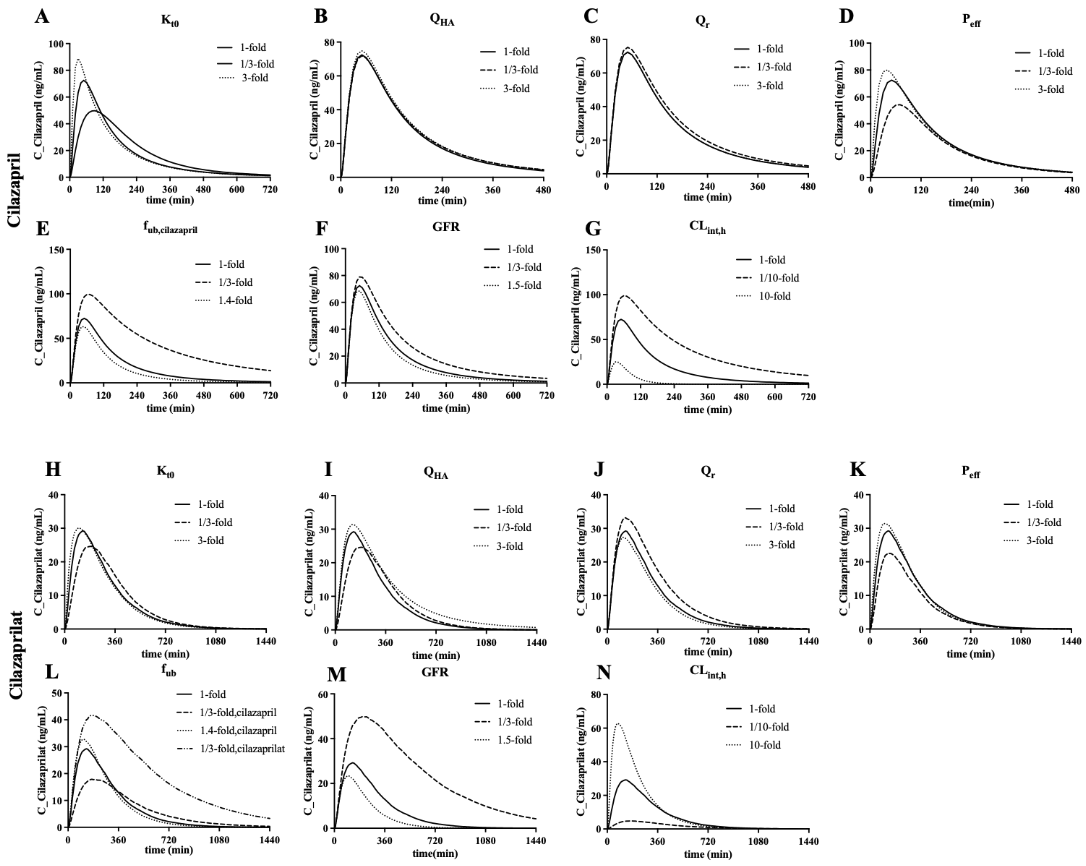

Figure S5. Sensitivity analysis following a single 2.5mg oral dose of cilazapril administered to adults. Effect of changes in K_{t0} (A), Q_{HA} (B), Q_r (C), P_{eff} (D), f_{ub} (E), GFR (F) and $CL_{int,h}$ (G) on plasma cilazapril; Effect of changes in K_{t0} (H), Q_{HA} (I), Q_r (J), P_{eff} (K), f_{ub} (L), GFR (M) and $CL_{int,h}$ (N) on plasma cilazaprilat.

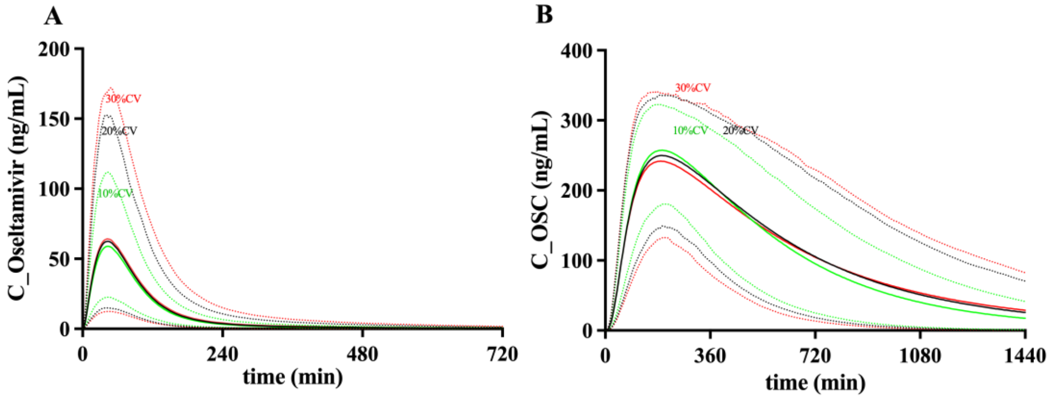

Figure S6. Impact of different coefficient of variation (CV) assumptions on simulated concentration-time profiles of oseltamivir and oseltamivir carboxylate (OSC). Solid lines represent the mean predicted concentration-time profiles, and dotted lines indicate the corresponding 5th–95th percentile prediction intervals. Different colors represent different CV assumptions (green: 10% CV; black: 20% CV; red: 30% CV).

References

1. Rodgers, T.; Rowland, M. Physiologically Based Pharmacokinetic Modelling 2: Predicting the Tissue Distribution of Acids, Very Weak Bases, Neutrals and Zwitterions. *J. Pharm. Sci.* **2006**, *95*, 1238–1257. doi:10.1002/jps.20502.
2. Russell, T. L., Berardi, R. R., Barnett, J. L., Dermentzoglou, L. C., Jarvenpaa, K. M., Schmaltz, S. P., Dressman, J. B. Upper gastrointestinal pH in seventy-nine healthy, elderly, North American men and women. *Pharm. Res*. **1993**, 10, 187–196. doi:10.1023/a:1018970323716.
3. Vinarov, Z., Abdallah, M., Agundez, J. A. G., Allegaert, K., Basit, A. W., Braeckmans, M., Ceulemans, J., Corsetti, M., Griffin, B. T., Grimm, M., et al. Impact of Gastrointestinal Tract Variability on Oral Drug Absorption and Pharmacokinetics: An UNGAP review. *Eur. J. Pharm. Sci*. **2021**, 162, 105812. doi: org/10.1016/j.ejps.2021.105812.
4. Gertz, M.; Houston, J.B.; Galetin, A. Physiologically Based Pharmacokinetic Modeling of Intestinal First-Pass Metabolism of CYP3A Substrates with High Intestinal Extraction. *Drug Metab. Dispos.* **2011**, *39*, 1633–1642. doi:10.1124/dmd.111.039248.
5. Chang, H.P.; Kim, S.J.; Wu, D.; Shah, K.; Shah, D.K. Age-Related Changes in Pediatric Physiology: Quantitative Analysis of Organ Weights and Blood Flows. *AAPS J.* **2021**, *23*, 50. doi:10.1208/s12248-021-00581-1.
6. Björkman, S. Prediction of Drug Disposition in Infants and Children by Means of Physiologically Based Pharmacokinetic (PBPK) Modelling: Theophylline and Midazolam as Model Drugs. *Br. J. Clin. Pharmacol.* **2005**, *59*, 691–704. doi:10.1111/j.1365-2125.2004.02225.x.
7. Schlender, J.-F.; Meyer, M.; Thelen, K.; Krauss, M.; Willmann, S.; Eissing, T.; Jaehde, U. Development of a Whole-Body Physiologically Based Pharmacokinetic Approach to Assess the Pharmacokinetics of Drugs in Elderly Individuals. *Clin. Pharmacokinet.* **2016**, *55*, 1573–1589. doi:10.1007/s40262-016-0422-3.
8. Stader, F.; Siccardi, M.; Battegay, M.; Kinvig, H.; Penny, M.A.; Marzolini, C. Repository Describing an Aging Population to Inform Physiologically Based Pharmacokinetic Models Considering Anatomical, Physiological, and Biological Age-Dependent Changes. *Clin. Pharmacokinet.* **2019**, *58*, 483–501. doi:10.1007/s40262-018-0709-7.
9. Jittamala, P.; Pukrittayakamee, S.; Tarning, J.; Lindegardh, N.; Hanpithakpong, W.; Taylor, W.R.J.; Lawpoolsri, S.; Charunwattana, P.; Panapipat, S.; White, N.J.; et al. Pharmacokinetics of Orally Administered Oseltamivir in Healthy Obese and Nonobese Thai Subjects. *Antimicrob. Agents Chemother.* **2014**, *58*, 1615–1621. doi:10.1128/AAC.01786-13.
10. Abe, M.; Smith, J.; Urae, A.; Barrett, J.; Kinoshita, H.; Rayner, C.R. Pharmacokinetics of Oseltamivir in Young and Very Elderly Subjects. *Ann. Pharmacother.* **2006**, *40*, 1724–1730. doi:10.1345/aph.1H174.
11. Tarkiainen, E.K.; Backman, J.T.; Neuvonen, M.; Neuvonen, P.J.; Schwab, M.; Niemi, M. Carboxylesterase 1 Polymorphism Impairs Oseltamivir Bioactivation in Humans. *Clin. Pharmacol. Ther.* **2012**, *92*, 68–71. doi:10.1038/clpt.2012.13.
12. Brennan, B.J.; Davies, B.; Cirrincione-Dall, G.; Morcos, P.N.; Beryozkina, A.; Chappey, C.; Aceves Baldó, P.; Lennon-Chrimes, S.; Rayner, C.R. Safety, Tolerability, and Pharmacokinetics of Intravenous Oseltamivir: Single- and Multiple-Dose Phase I Studies with Healthy Volunteers. *Antimicrob. Agents Chemother.* **2012**, *56*, 4729–4737. doi:10.1128/AAC.00200-12.
13. Snell, P.; Dave, N.; Wilson, K.; Rowell, L.; Weil, A.; Galitz, L.; Robson, R. Lack of Effect of Moderate Hepatic Impairment on the Pharmacokinetics of Oral Oseltamivir and Its Metabolite Oseltamivir Carboxylate. *Br. J. Clin. Pharmacol.* **2005**, *59*, 598–601. doi:10.1111/j.1365-2125.2005.02340.x.
14. Rath, B.A.; Brzostek, J.; Guillén, S.; Niranjan, V.; Chappey, C.; Rayner, C.R.; Clinch, B. Safety, Virology and Pharmacokinetics of Oseltamivir in Infants with Laboratory-Confirmed Influenza: A Phase I/II, Prospective, Open-Label, Multicentre Clinical Trial. *Antivir. Ther.* **2015**, *20*, 815–825. doi:10.3851/IMP2967.

15. Kimberlin, D.W.; Acosta, E.P.; Prichard, M.N.; Sánchez, P.J.; Ampofo, K.; Lang, D.; Ashouri, N.; Vanchiere, J.A.; Abzug, M.J.; Abughali, N.; et al. Oseltamivir Pharmacokinetics, Dosing, and Resistance among Children Aged <2 Years with Influenza. *J. Infect. Dis.* **2013**, *207*, 709–720. doi:10.1093/infdis/jis765.
16. Tang Girdwood, S.C.; Mizuno, T.; Krallman, K.A.; Benken, L.A.; Stoneman, E.M.; Yunger, T.M.; Wong, H.R.; Vinks, A.A.; Kaplan, J.M. Route of Oseltamivir Administration Affects Metabolite Concentrations in Critically Ill Children. *Infect. Dis. J.* **2019**, *38*, 1224–1227. doi:10.1097/INF.0000000000002473.
17. Bautista, F.; Engelhard, D.; Rizzari, C.; Baka, M.; Saavedra-Lozano, J.; Lopez-Medina, E.; Nasmyth-Miller, C.; Hernández-Sánchez, J.; Sturm, S. Pharmacokinetics and Pharmacodynamics of Conventional-Dose vs Triple-Dose Oseltamivir in Severely Immunocompromised Children With Influenza. *Open Forum Infect. Dis.* **2019**, *6*, ofz430. doi:10.1093/ofid/ofz430.
18. Massarella, J.W.; He, G.Z.; Dorr, A.; Nieforth, K.; Ward, P.; Brown, A. The Pharmacokinetics and Tolerability of the Oral Neuraminidase Inhibitor Oseltamivir (Ro 64–0796/GS4104) in Healthy Adult and Elderly Volunteers. *J. Clin. Pharmacol.* **2000**, *40*, 836–843. doi:10.1177/00912700022009567.
19. Ohnishi, A.; Tsuboi, Y.; Ishizaki, T.; Kubota, K.; Ohno, T.; Yoshida, H.; Kanezaki, A.; Tanaka, T. Kinetics and Dynamics of Enalapril in Patients with Liver Cirrhosis. *Clin. Pharmacol. Ther.* **1989**, *45*, 657–665. doi:10.1038/clpt.1989.87.
20. Marzo, A.; Bo, L.; Mazzucchelli, P.; Monti, N.; Crivelli, F.; Ismaili, S.; Giusti, A.; Uhr, M. Pharmacokinetic and Pharmacodynamic Comparative Study of Zofenopril and Enalapril in Healthy Volunteers. *Arzneimittelforschung* **2002**, *52*, 233–242. doi:10.1055/s-0031-1299886.
21. Baba, T.; Murabayashi, S.; Tomiyama, T.; Takebe, K. The Pharmacokinetics of Enalapril in Patients with Compensated Liver Cirrhosis. *Br. J. Clin. Pharmacol.* **1990**, *29*, 766–769. doi:10.1111/j.1365-2125.1990.tb03700.x.
22. Moffett, B.S.; DiSanto, A.R.; Espinosa, O.; Hou, J.; Colabuono, P. Bioequivalence of Enalapril Oral Solution for Treatment of Pediatric Hypertension and Enalapril Tablets. *Clin. Pharm. Drug Dev.* **2014**, *3*, 493–498. doi:10.1002/cpdd.129.
23. Portolés, A.; Terleira, A.; Almeida, S.; García-Arenillas, M.; Caturla, M.-C.; Filipe, A.; Vargas, E. Bioequivalence Study of Two Formulations of Enalapril, at a Single Oral Dose of 20 Mg (Tablets): A Randomized, Two-Way, Open-Label, Crossover Study in Healthy Volunteers. *Curr. Ther. Res.* **2004**, *65*, 34–46. doi:10.1016/S0011-393X(04)90003-3.
24. Hersh, A. D., Kelly, J. G., Laher, M. S., Carmody, M., Doyle, G. D. Effect of Hydrochlorothiazide on the Pharmacokinetics of Enalapril in Hypertensive Patients with Varying Renal Function. *J. Cardiovasc. Pharmacol.* **1996,** 27(1), 7–11. doi: 10.1097/00005344-199601000-00002.
25. Macdonald, N.; Sioufi, A.; Howie, C.; Wade, J.; Elliott, H. The Effects of Age on the Pharmacokinetics and Pharmacodynamics of Single Oral Doses of Benazepril and Enalapril. *Br. J. Clin. Pharmacol.* **1993**, *36*, 205–209. doi:10.1111/j.1365-2125.1993.tb04218.x.
26. Hockings, N.; Ajayi, A.; Reid, J. Age and the Pharmacokinetics of Angiotensin Converting Enzyme Inhibitors Enalapril and Enalaprilat. *Br. J. Clin. Pharmacol.* **1986**, *21*, 341–348. doi:10.1111/j.1365-2125.1986.tb05205.x.
27. Weisser, K.; Schloos, J.; Jakob, S.; Mühlberg, W.; Platt, D.; Mutschler, E. The Influence of Hydrochlorothiazide on the Pharmacokinetics of Enalapril in Elderly Patients. *Eur. J. Clin. Pharmacol.* **1992**, *43*, 173–177. doi:10.1007/BF01740666.
28. Wells, T.; Rippley, R.; Hogg, R.; Sakarcan, A.; Blowey, D.; Walson, P.; Vogt, B.; Delucchi, A.; Lo, M.; Hand, E.; et al. The Pharmacokinetics of Enalapril in Children and Infants with Hypertension. *J. Clin. Pharmacol.* **2001**, *41*, 1064–1074. doi:10.1177/00912700122012661.
29. Williams, P.E.O.; Brown, A.N.; Rajaguru, S.; Francis, R.J.; Walters, G.E.; McEwen, J.; Durnin, C. The Pharmacokinetics and Bioavailability of Cilazapril in Normal Man. *Br. J. Clin. Pharmacol.* **1989**, *27*, 181S–188S. doi:10.1111/j.1365-2125.1989.tb03480.x.

30. Massarella, J.; DeFeo, T.; Lin, A.; Limjuco, R.; Brown, A. The Pharmacokinetics and Dose Proportionality of Cilazapril. *Br. J. Clin. Pharmacol.* **1989**, *27*, 199S–204S. doi:10.1111/j.1365-2125.1989.tb03482.x.
31. Nilsen, O.G.; Sellevold, O.F.M.; Romfo, O.S.; Smedsrud, A.; Grynne, B.; Williams, P.E.O.; Kleinbloesem, C.H. Pharmacokinetics and Effects on Renal Function Following Cilazapril and Hydrochlorothiazide Alone and in Combination in Healthy Subjects and Hypertensive Patients. *Br. J. Clin. Pharmacol.* **1989**, *27*, 323S–328S. doi:10.1111/j.1365-2125.1989.tb03499.x.
32. Williams, P.E.O.; Brown, A.N.; Rajaguru, S.; Walters, G.E.; McEwen, J.; Durnin, C. A Pharmacokinetic Study of Cilazapril in Elderly and Young Volunteers. *Br. J. Clin. Pharmacol.* **1989**, *27*, 211S–215S. doi:10.1111/j.1365-2125.1989.tb03484.x.
33. Anderson, P.J.; Critchley, J.A.J.H.; Tomlinson, B. A Comparison of the Pharmacokinetics and Pharmacodynamics of Cilazapril between Chinese and Caucasian Healthy, Normotensive Volunteers. *Eur. J. Clin. Pharmacol.* **1996**, *50*, 57–62. doi:10.1007/s002280050069.
